# Arterial Hypertension and Unusual Ascending Aortic Dilatation in a Neonate With Acute Kidney Injury: Mechanistic Computer Modeling

**DOI:** 10.3389/fphys.2019.01391

**Published:** 2019-11-08

**Authors:** Luis Altamirano-Diaz, Andrea D. Kassay, Baran Serajelahi, Christopher W. McIntyre, Guido Filler, Sanjay R. Kharche

**Affiliations:** ^1^Department of Paediatrics, Schulich School of Medicine and Dentistry, Western University, London, ON, Canada; ^2^Children’s Health Research Institute, London, ON, Canada; ^3^Paediatric Cardiopulmonary Research Laboratory, LHSC, London, ON, Canada; ^4^Lawson Health Research Institute, London, ON, Canada; ^5^Department of Medicine, Schulich School of Medicine and Dentistry, Western University, London, ON, Canada; ^6^Department of Medical Biophysics, Western University, London, ON, Canada

**Keywords:** dialysis, hypertension, aortic dilatation, computer model, lumped parameter blood flow model, sensitivity analysis

## Abstract

**Background:**

Neonatal asphyxia caused kidney injury and severe hypertension in a newborn. An unusually dilatated ascending aorta developed. Dialysis and pharmacological treatment led to partial recovery of the ascending aortic diameters. It was hypothesized that the aortic dilatation may be associated with aortic stiffening, peripheral resistance, and cardiovascular changes. Mathematical modeling was used to better understand the potential causes of the hypertension, and to confirm our clinical treatment within the confines of the model’s capabilities.

**Methods:**

The patient’s systolic arterial blood pressure showed hypertension. Echocardiographic exams showed ascending aorta dilatation during hypertension, which partially normalized upon antihypertensive treatment. To explore the underlying mechanisms of the aortic dilatation and hypertension, an existing lumped parameter hemodynamics model was deployed. Hypertension was simulated using realistic literature informed parameter values. It was also simulated using large parameter perturbations to demonstrate effects. Simulations were designed to permit examination of causal mechanisms. The hypertension inducing effects of aortic stiffnesses, vascular resistances, and cardiac hypertrophy on blood flow and pressure were simulated. Sensitivity analysis was used to stratify causes.

**Results:**

In agreement with our clinical diagnosis, the model showed that an increase of aortic stiffness followed by augmentation of peripheral resistance are the prime causes of realistic hypertension. Increased left ventricular elastance may also cause hypertension. Ascending aortic pressure and flow increased in the simultaneous presence of left ventricle hypertrophy and augmented small vessel resistance, which indicate a plausible condition for ascending aorta dilatation. In case of realistic hypertension, sensitivity analysis showed that the treatment of both the large vessel stiffness and small vessel resistance are more important in comparison to cardiac hypertrophy.

**Conclusion and Discussion:**

Large vessel stiffness was found to be the prime factor in arterial hypertension, which confirmed the clinical treatment. Treatment of cardiac hypertrophy appears to provide significant benefit but may be secondary to treatment of large vessel stiffness. The quantitative grading of pathophysiological mechanisms provided by the modeling may contribute to treatment recommendations. The model was limited due to a lack of data suitable to permit model identification.

## Introduction

### Clinical Background

Ascending aortic dilatation and arterial hypertension may be related to an increased aortic blood vessel stiffening (Milan et al., 2013). Aortic dilatation has recently been identified as a frequent complication of chronic kidney disease (Kaddourah et al., 2015). Patients with kidney injury often have simultaneous aortic abnormalities, as well as peripheral and pulmonary vasculature abnormalities. In this study, we present the development of severe but reversible aortic dilatation in a neonate with acute kidney injury. The patient developed arterial hypertension owing to volume overload despite dialysis. We clinically hypothesized that the aortic dilatation may be, at least partially, caused by the arterial (aortic) hypertension, increased aortic stiffening, increased cardiac hypertrophy, and an augmented peripheral small vessel resistance. Our hypothesis is supported by previous studies. It has been shown that arterial hypertension increases aortic wall stress leading to an increase of the ascending aorta’s luminal diameter (Rabkin and Janusz, 2013). Specifically, the hypertension induced high systolic blood pressure likely increased the aorta’s diameter (Hardt et al., 1999). Further, a recent experimental-computational study (Pettersen et al., 2014) has shown that arterial stiffness is an independent and sufficient condition to explain hypertension. Whereas it is now established that augmentation of peripheral microvascular resistance may promote hypertension (Serne et al., 2007), cardiac hypertrophy is also closely associated with hypertension (Kahan, 1998). Thus, both peripheral microvasculature resistance and cardiac hypertrophy may also contribute to aortic dilatation. Mechanistic computer modeling of whole body hemodynamics was deployed to stratify the relative importance of these four factors, and uncover other unknowns.

### Biophysical Modeling Background

*In silico* biophysical modeling is now an established diagnostic-prognostic methodology which is increasingly assisting clinicians in their practice (Marsden, 2013). Recent studies by Kharche et al. (2017, 2018) demonstrate the application of modeling for a mechanistic interpretation and integration of observational (experimental, imaging, and clinical) data to enhance knowledge and understanding. Mathematical models permit integration of the multiple individual processes, and to simulate emergent behavior in complex biological systems such as whole-body hemodynamics. Mathematical modeling is an ideal tool to analyze the roles of individual processes on the complex system’s emergent behavior. Extant whole body hemodynamics models deploy a multi-scale methodology that comprises of a hybrid lumped parameter (0D) to spatially extended (1D and 3D) components that can be personalized to patient measurements (Marsden and Moghadam, 2015; Caruso et al., 2016). Whereas spatially extended (1D, 2D, and 3D) modeling permits detailed computation of relevant quantities such as velocity profiles and wall shear, they remain computationally intensive. In contrast, 0D models based on Windkessel elements are themselves powerful tools that incorporate blood vessel resistances, stiffness, and flow reserve (see Supplementary Table S1 for electrical-hemodynamic equivalence of components), and are relatively computationally tractable (Korakianitis and Shi, 2006b; Heldt et al., 2010; Shi et al., 2011). The presented numerical results are based on the 0D model by Korakianitis and Shi (2006a). It was used because it is validated using clinical data and is suitable for the scope of this study. The model provides biophysical relationships between hypertension defining aortic pressure (and blood flow) and multiple types of blood vessel resistances, stiffnesses, as well as cardiac properties such as wall elastance. The model permits approximate reproducing of observed clinical data in terms of blood vessel properties and high aortic pressure, i.e., hypertension. Finally, the model permits identifying the important targets for treatments in terms of blood vessel and cardiac properties.

### Computational Model Aims

In line with our clinical hypothesis, the aims of the modeling part were to:

i Identify the most significant hypertension and aortic dilatation promoting mechanisms in the model (Korakianitis and Shi, 2006a); and
ii identify potential cardiac causes of the observed ascending aortic dilatation.

## Case Report

### Ethics Statement

This case report conforms to the Declaration of Helsinki. The patient data was obtained during routine clinical examinations. As less than six patients were examined, this case report was exempt from ethics approval. Written informed consent to use the neonate patient’s data in, and publication of, this case report was obtained from the patient’s parent. All patient data was anonymized prior to use in this work.

### Relevant Patient Data

**FIGURE 1:**
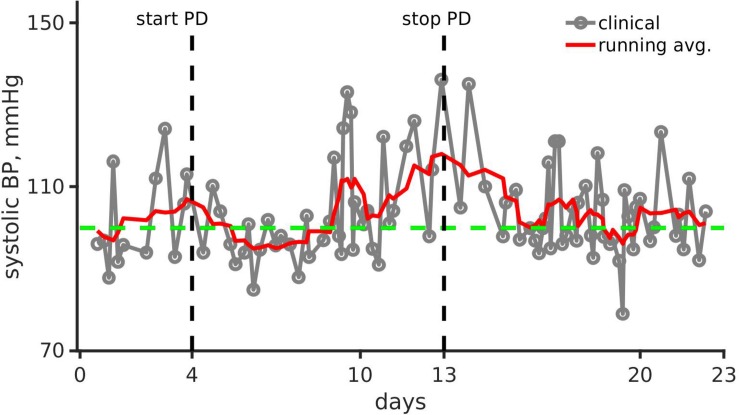
Clinical observation and running mean of systolic blood pressure (mmHg) over the first 23 days of the pediatric patient’s life. Gray line joined points show the clinical recording. The running mean is shown as a solid red line. The green dashed line shows systolic blood pressure (BP) for healthy infants (Fleming et al., 2011). Black vertical dashed lines show day of peritoneal dialysis (PD) onset (left, day 4) and end (right, day 13).

**FIGURE 2:**
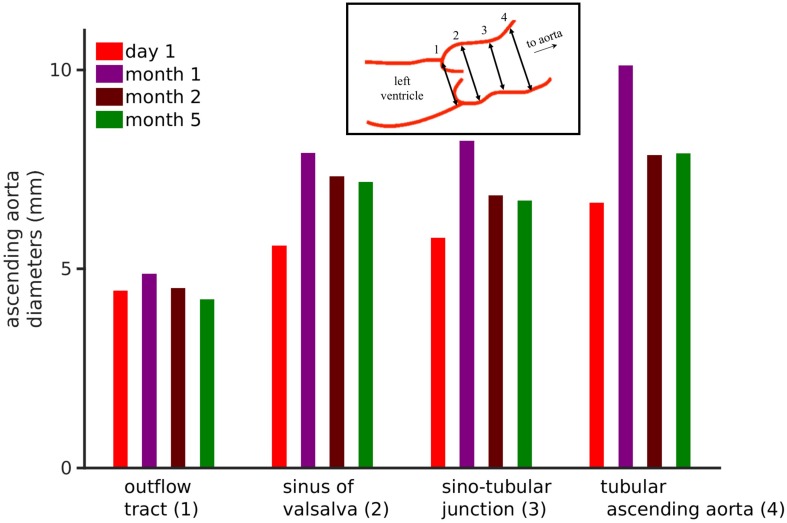
Echocardiographic data of the patient’s ascending aortic diameters. Inset shows the four aortic diameters that were measured, i.e., outflow tract (1), sinuses of valsalva (2), sinotubular junction (3), and tubular ascending aorta (4). The bar chart shows the four ascending aortic diameters over a 6-month period. See Supplementary Figure S2 for frame-by-frame temporal data.

The patient’s birth was complicated. She developed acute kidney injury due to systemic water retention (hypotension or volume overload) by day 2 of life. She developed arterial hypertension, which was likely because of volume overload (see below, and Supplementary Section S1.1). Her ascending aorta was observed to be dilatated, which was also a major clinical concern. Whereas our diagnostics eliminated the possibilities of Marfarn’s syndrome as well as Loyes-Dietz syndrome, commencement of lifesaving treatment was prioritized over time consuming diagnosis of other potentially secondary conditions (see section “Discussion”). The volume overload was treated using peritoneal dialysis commencing on day 5 of life and continued for 9 days. To treat the aortic stiffness and peripheral resistance and associated reactions, first a calcium channel blocker (Amlodipine) and later an angiotensin converting enzyme inhibitor (enalapril) were prescribed and continued throughout the period of her observation and treatment. Figure 1 shows the systolic blood pressure in the first month of life. Echocardiogram exam performed at day 1 of life showed normal cardiac structure and normal ascending aortic diameter. The repeated echocardiogram exam at 1 month of age showed significant ascending aortic dilatation (Figure 2). The echocardiogram exam at month 5 showed aortic dilatation to be considerably reduced. A full description of the case report is given in the Supplementary Section S1.1. Representative echo frames are show in Supplementary Figure S1. Diameters over one cardiac cycle are shown in Supplementary Figure S2.

## Computational Methods

### Clinical Data Interpretation, Blood Pressure Measurements

Figure 1 shows the patient’s smoothed systolic blood pressure along with the systolic blood pressure of a healthy neonate (100 mmHg).

### Clinical Data Interpretation, Ascending Aortic Diameter Assessment

Parasternal long-axis view echo exams were used to measure ascending aortic diameters. The frame rates were between 50 and 91 frames per minute. Echo exams are performed to acquire images over three heart beats. Four echo exams were recorded each at day 1, day 30, day 60, and day 150. The pixel size in the echo images was DX = 0.075 mm and DY = 0.075 mm. From each exam, individual frames for the duration of 1 cardiac cycle were extracted. In each exam, four diameters (Figure 2, inset) namely the outflow tract, sinuses of Valsalva, sinotubular junction, and tubular ascending aorta diameters were measured according to clinical recommendations (Evangelista et al., 2010; Flachskampf et al., 2014). The measurements were performed using our semi-automatic segmentation method based on ImageJ-FiJi software (Schindelin et al., 2012). Details of the segmentation method are provided in Supplementary Section S1.2.

### Biophysical Model Implementation

The model (Korakianitis and Shi, 2006a) consists of coupled hemodynamic inter-relationships described by ordinary differential equations, and was obtained from CellML repository (Beard et al., 2009). The model equations were implemented in both MATLAB and C languages. In C language, the equations were solved using our in house robust implicit solvers as described previously (Kharche et al., 2014, 2017). Preliminary simulations were performed using the MATLAB version to prototype and assess overall behavior. The numerically efficient C version was used in the larger parameter sweep simulations. The model codes and anonymized clinical data are freely available at the repository: https://github.com/mccsssk2/Arterial-Hypertension.

### Simulation Protocols

The model (illustrated in Supplementary Figure S3) consists of a systemic loop, pulmonary loop, and representations of the heart chambers and corresponding valves. The hemodynamic loops consist of various large and small blood vessels that are assigned physiological resistances, compliances, and inductances. The heart has four chambers and uni-directional blood flow valves. Each chamber pumps blood to the next downstream component of the model. The pumping strength of each chamber is summarized by its elastance, E. In particular, the left ventricular elastance, E_*lv*_, oscillates between a maximum (E_*lv,max*_) and minimum (E_*lv,min*_) values, this pumping action leads to an oscillating left ventricular pressure as shown in Supplementary Figure S4Ai.

In all simulations, parameter values were chosen for specific experiments and 1000 s of hemodynamics activity simulated for each instance of parameter values, which provided steady dynamics that were independent of initial conditions. The model was verified to have achieved steady dynamics, either steady state or steady oscillations, between 980 and 1000 s. The final 20 s of data were analyzed to generate results. Multiple simulations were performed to ascertain the effect of perturbing each parameter, which informed the results presented in this work.

#### Simulation Parameters’ Biophysical Meaning

Relevant model parameters were assigned the following interpretation within the scope of this study. The systemic capillary bed resistance, R_*SCP*_, and systemic arterioles resistance, R_*SAR*_, represented small artery resistances. These small vessel arterial resistances represent kidney injury induced alteration of peripheral microvascular resistances. As the model does not functionally distinguish between R_*SCP*_ and R_*SAR*_, they may be interpreted interchangeably. The model incorporates compliances of all major blood vessels. Compliance is inversely proportional to blood vessel stiffness. In this study, the major compliances that we considered were systemic artery (i.e., aorta) compliance, C_*SAT*_; vena cava compliance, C_*SVN*_; and pulmonary vein compliance, C_*PVN*_. These compliances of the large blood vessels permitted simulation of aortic blood vessel stiffening, as well as stiffening of vena cava and pulmonary veins. The elastance of the left ventricle, E_*lv*_, oscillates between prescribed minimum (E_*lv,min*_) and maximum (E_*lv,max*_) values in the model. To simulate hypertrophy, the left ventricular pumping function, E_*lv,max*_, was increased.

#### Simulation Outputs

For each instance of every numerical simulation experiment, all model outputs as time profiles were recorded. Baseline time profiles for a few heart beats are shown in Supplementary Figure S4. The time profiles were then analyzed to characterize model behavior in terms of systolic and diastolic values. The following model behavior was characterized by extracting systolic and diastolic values of pressures and blood flows from the time profiles. Systemic artery (aorta) blood pressure, P_*SAT*_, was recorded to characterize arterial hypertension. The pulmonary artery pressure, P_*PAT*_, was used to characterize effects of hypertension inducing factors on the pulmonary circulation. Reduced renal function may simultaneously reduce blood flow in the vena cava and urine amounts. An explicit excretion process is unavailable in the model. In the event of increased renal arterial microvascular resistance, the draining venous (i.e., systemic vein interpreted as vena cava) flow may be reduced and be at a significantly lower pressure. Due to the structure of our model, an increased peripheral (which includes renal) microvascular resistance may be expected to reduce both quantities. Urine output was therefore characterized using vena cava flow, Q_*SVN*_. In addition to P_*SAT*_, the systemic artery blood flow, Q_*SAT*_, was recorded to characterize the effects of hypertension on aortic flow. Among the wide spectrum of clinically significant conditions causing pediatric aortic dilatation (Aalberts et al., 2008), our young patient tested negatively for structural remodeling and did not have either Marfarn Syndrome nor Loyes-Dietz Syndrome (see section “Case Report”, Supplementary Section S1.1). However, the absence of clinically significant structural remodeling may not completely rule out the interplay between mechanical dysfunction and aortic wall’s deterioration (see section “Discussion”). For the purposes of the present modeling, we assumed an absence of structural remodeling based on the clinical diagnosis, and hypothesized that dilatation was potentially regulated by the local pressure, or flow, or both. Whereas the flow along the ascending aorta is already defined in the model, Q_*AO*_, the pressure at the ascending aorta was taken to be P_*SAT*_. The left ventricle pressure, P_*lv*_, and volume, V_*lv*_, were recorded to construct pressure-volume loops. Areas of pressure-volume loops represent cardiac stroke work and were used to compare cardiac status under normal, hypertensive, hypertrophied, and other conditions. Baseline model behavior is illustrated in Supplementary Figure S4. Dynamical variables used in this study are given in Supplementary Table S2. All dynamical variables had nil initial conditions. Model parameters values used in this study are listed in Supplementary Table S3.

### Modeling Definitions of the Clinical Conditions

Following definitions of the clinical conditions within the scope of the model were used. The volume overload due to the acute kidney injury, as well as autoregulatory responses such as to the renin-angiotensin-aldosterone system were assumed to increase systemic microvascular resistances, R_*SAR*_ and R_*SCP*_. Compliances (C_*SAT*_, C_*SVN*_, and C_*PVN*_) were reduced to simulate increase of stiffness. The effect of an increased heart rate was simulated by altering the pacing period of the heart as described in the model (Korakianitis and Shi, 2006a, b). Finally, it is also known that kidney injury may cause cardiac hypertrophy. Left ventricle hypertrophy was simulated by increasing the maximum elastance, E_*lv*_, as described in the model (Korakianitis and Shi, 2006a, b). Multiple outputs characterized hypertension, venous flow status, pulmonary sufficiency, and ascending aortic size. Increase of the systemic artery systolic pressure (maximum of P_*SAT*_ waveform) was defined as hypertension. Whereas the clinically accepted definition of hypertension encompasses multiple factors, the data informing this study was that of the patient’s arterial systolic blood pressure. Simultaneous increase of the flow through the aortic valve, Q_*AO*_, and the left ventricle systolic pressure, P_*LV*_, was assumed to be directly proportional to ascending aortic diameters. The flows in the systemic vein (or vena cava), Q_*SVN*_, pulmonary artery, Q_*PAT*_, as well as the pressure in the pulmonary artery, P_*PAT*_, were used to assess status of venous flow and pulmonary sufficiency. The left ventricle pressure, P_*lv*_, and volume, V_*lv*_, were recorded to construct pressure-volume loops. Areas of pressure-volume loops represent cardiac stroke work and were used to compare cardiac status under normal, hypertensive, hypertrophied, and other conditions.

### First Order Dynamic Local Sensitivity Analysis

Sensitivity analysis is a mathematical method to assess the impact of altering parameters (e.g., arterial microvascular resistance, R_*SAR*_) on model output (e.g., aortic pressure, P_*SAT*_). Dynamic sensitivity analysis was used to assess impact of parameter alterations on time varying model output. Whereas global sensitivity measures provide information regarding overall model metrics (Kharche et al., 2009), the present model was analyzed using dynamic sensitivity measures (Saltelli, 2002; Ferretti et al., 2016). Within the scope of this study, disease and treatments are simulated as changes of model parameters such as blood vessel resistances and stiffness. For instance, aortic stiffening may be more important in causing arterial hypertension as compared to an increase of microvascular resistances as the patient’s status altered from healthy (baseline) to diseased. The impact of parameters on model outputs is defined by absolute values of parameters, which represent the baseline or diseased conditions of the patient. Under diseased conditions of augmented microvascular resistances, aortic stiffness may also affect aortic pressure significantly. Therefore, changes of a set of parameters may be relevant in generating disease phenotype, but a different set of parameters may have to be considered in the treatment options.

The sensitivity analysis was designed to demonstrate the effectiveness of anti-hypertensive treatments. The sensitivity of model outputs to modeling parameters was computed using established methods (Gul and Bernhard, 2015; Gul et al., 2016). The definition of the dynamic sensitivity index is derivative based. For a given variable *V*_*i*_, and a parameter *p*_*j*_, the sensitivity of *V*_*i*_ to *p*_*j*_ at a given parameter value *p*_*j,o*_ is defined as

(1) $$S_{i\,j}=p_{j}\frac{\partial V_{i}}{\partial p_{j}}$$

The indices *S*_*ij*_ were computed using finite difference based direct differentiation. *S*_*ij*_ were computed for each (*V_i_*, *p_j_*) combination of interest. As *S*_*ij*_ is local, it was computed under control and pathological conditions to permit assessment of the changes in sensitivity of relevant variables *V*_*i*_ to *p*_*j*_ at different values (*p_j,o1_*, *p_j,o2_*, …), thus permitting to assess the maximum effectiveness of a given treatment in a range of parameter values. To the best of our knowledge, quantitative data regarding alterations of model parameters under pathological conditions is scarce. We reiterate that our aim was to indicatively simulate the effects of hypertension, and as such, the results are phenomenological. We first identified parameter values to provide a definition of realistic hypertension (hypertension 1, h1). Cohen-Solal et al. (1994) estimated left ventricular elastance in a normotensive group of patients as well as in three groups of hypertensive patients, they found that mean E_*lv*_ was approximately 200% higher in the normotensive group when compared to the mean E_*lv*_ across all there hypertensive groups. When investigating the relationship between aortic compliance and human hypertension (Liu et al., 1989) reported that aortic compliance was 53% lower in their hypertensive group when compared to their normotensive group. Finally, hypertension has been associated with a 10% increase in renal resistive index as described by Galesic et al. (2000). Further, the heart periods are known to range between 0.5 to 1.5 s in hypertensive patients (Boegehold et al., 1991). In light of these results, a literature motivated diseased condition was simulated by increasing R_*SAR*_ by 50%, reducing C_*SAT*_ to 0.47 of its baseline value, and increasing E_*lv*_ by twofold. This literature motivated case is referred to as hypertension 1. To more markedly show the effects of perturbing these parameters and to explore the wider parameter space, hypertension (hypertension 2, h2) was also simulated using their exaggerated values. The diseased condition was simulated by increasing R_*SAR*_ by 25-fold, reducing C_*SAT*_ (to simulate increased arterial stiffness) to 0.25 of its baseline value, and increasing E_*lv*_ by tenfold (Boegehold et al., 1991; Abdelhammed et al., 2005). Although the chosen parameter values for hypertension 2 are arbitrary, a particular patient’s condition could be deduced from presented results. Each hypertensive state was simulated at two heart rates. A high heart rate of 2 Hz was used to simulate the patient’s diseased condition. A more physiological heart rate of 1 Hz was also used. The implementation of these hypertension inducing alterations was deemed to partially serve as a model of the patient’s status. The interpretation of the sensitivity indices, *S*_*ij*_, was performed as follows. *S*_*ij*_ curves of a variable *V*_*i*_ with respect to a parameter *p*_*j*_ in the baseline and pathological state were compared. If *S*_*ij*_ was found to be positive, *V*_*i*_ was deemed to increase as *p*_*j*_ increased. If *S*_*ij*_ was negative, then *V*_*i*_ was deemed to reduce as *p*_*j*_ increased. If *S*_*ij*_ is larger than *S_ik_*, under pathological conditions in comparison to baseline conditions, then treatment that brought parameter *p*_*j*_ to physiological values would be preferred over other treatments that altered *p*_*k*_, *k* ≠ *j*.

## Results, Clinical Observations

As shown in Figure 1, the systolic arterial blood pressure increased despite dialysis. However, the pressure normalized within a month after a continuous combination therapy of amlodipine (0.24 mg/kg/day, dosing range is 0.1–0.5 mg/kg/day) and enalapril (0.15 mg/kg/day, dosing range is 0.1–0.5 mg/kg/day). The home blood pressure readings show significantly reduced hypertension, and were within the 50th percentile (Flynn et al., 2008).

The neonate’s first echocardiogram at day one showed normal tri-leaflet aortic value, a normal mitral valve with no evidence of mitral valve prolapse, two small muscular ventricular septal defects, normal left ventricular mass 45 g/m^2^, and normal ascending aortic diameter 0.65 cm with a Z score of 0.95 (Supplementary Figure S1A; Warren et al., 2006). Echocardiogram was repeated at 1 month of life, which showed increased left ventricle mass 105.3 g/m^2^, ascending aortic dilatation 1.01 cm with a Z score of 4.88 (Supplementary Figure S1B). At 2 months of age, the ascending aortic diameter was 0.78 cm, at 5 months of age the aortic diameter remained at 0.78 cm (Z score 2.81) and 12 months the ascending aortic diameter was normal for weight and height measuring 1.4 cm (Z score 1.37). As the dimensions of the heart constantly change in a growing child, cardiac dimensions are compared using age-independent standard deviation scores or Z scores. Further analysis of the echo images (Figure 2) show four standard ascending aortic diameters. Each of the diameters increased at 1 month. After 1 month and up to 6 months, all diameters consistently reduced closer toward physiological values. Representative echocardiography images are presented in Supplementary Figure S1. At last follow up, the aortic dimensions returned to the normal range (data not shown).

Whereas the clinical data did not constitute quantitative inputs to the model, it phenomenologically informed the modeling. The systolic arterial blood pressure was taken to represent hypertension and assumed to represent systemic artery (aortic) systolic pressure. The echocardiographic data was taken to represent events at the ascending aorta.

## Modeling Results

### Interpretation of the Clinical Data, Systolic Blood Pressure Recordings

As shown in Figure 1, the systolic blood pressure changed significantly over the period of the month. Immediately after dialysis and pharmacological treatment onset, the systolic blood pressure reduced markedly. Systolic blood pressure increased by over 27% from 90 mmHg (day 1, normal range 67–84 mmHg) to 115 mmHg (day 15, normal range 72–104 mmHg) showing hypertension. With anti-hypertensive treatment, the systolic blood pressure reduced to around 105 mmHg (normal range 72–104 mmHg) after dialysis was stopped. Therefore, we identified potential parameters that could give rise to hypertension, and alteration of parameters that could reduce the hypertension. The two parameter sets may not be the same.

### Interpretation of the Clinical Data, Ascending Aortic Diameters Over First Six Months of Patient’s Life

Analysis of the echocardiography based four ascending aorta diameters (Evangelista et al., 2010; Flachskampf et al., 2014) per cardiac cycle is shown in Figure 2. The corresponding frame-by-frame variation of the same diameters over a few cardiac cycles is shown in Supplementary Figure S2. The outflow tract, which is the closest to the left ventricle, showed an increase in its diameter after which it reduced. In comparison, the diameters measured further away from the ventricle increased over the course of 1 month and recovered partially by month 5. However, the dialysis and anti-hypertensive treatments can be seen to be effective as all aortic diameters reduced significantly after month 1 of the patient’s life. Because of the treatments, the outflow tract diameter reduced by 5% at month 5, while the diameters of sinus of Valsalva, sinotubular junction, and tubular ascending aorta remained at 25.1, 14.8, and 17.1% higher as compared to day 1 diameters. A negative change signifies a reduction of diameter at the end of dialysis-antihypertensive therapies, whereas a positive change signifies sub-optimal reduction of diameter. Therefore, the effect of altering aortic flow (which is directly related to aortic diameter) on model simulated pressures and blood flows in the body was explored.

### Interpretation of the Clinical Data, Other Measurements

During dialysis, the patient’s urine output was alarmingly negligible. As shown in Supplementary Figure S7A, the patient was under weight at birth (Meyers et al., 2013). She also had a high heart rate (Supplementary Figure S7B), in contrast to the development in healthy children where the heart rate decreases during the first few months of life (Fleming et al., 2011). These observations led us to investigate the causes of systolic blood pressure and reduced venous blood flow. The roles of hypertension inducing factors on pulmonary circulation were also examined using the model, to test its predictive capability. Finally, the model was used to identify the cardiovascular causes of aortic dilatation.

### Baseline Model

The model’s baseline behavior is reproduced in Supplementary Figure S4. The left ventricle and systemic artery (i.e., aorta) generate a systolic blood pressure of 120 mmHg (Supplementary Figure S4Ai). The systemic vein has a blood pressure of approximately 15 mmHg (Supplementary Figure S4Ai). The pulmonary artery has a systolic blood pressure of 50 mmHg in the model, but a much higher diastolic blood pressure compared to the right ventricle (Supplementary Figure S4Aii). The pulmonary vein, in contrast, has a markedly low pressure as well as oscillation amplitude (gray line in Supplementary Figure S4Aii). The blood flows in the left ventricle, systemic artery (aorta), and systemic vein (vena cava) are shown in Supplementary Figure S4Bi, and of the right ventricle, pulmonary artery, and pulmonary vein in Supplementary Figure S4Bii. The model baseline behavior was further characterized by the pressure-volume loop and its area (Supplementary Figure S5).

### Peripheral Microvascular (Small Systemic Blood Vessel) Resistance and Large Vessel Stiffness Cause Hypertension

**FIGURE 3:**
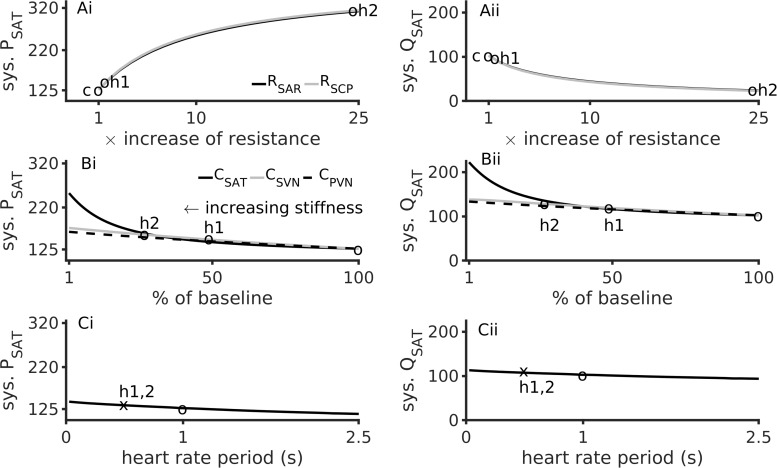
Major factors inducing hypertension. Left column shows data for aortic systolic pressure, sys. P_*SAT*_. Right column shows data for the aorta’s systolic blood flow, sys. Q_*SAT*_. Top row **(Ai,Aii)** shows data for effects of augmented peripheral resistance, middle row **(Bi,Bii)** shows data for effects of augmented arterial stiffnesses, and bottom row **(Ci,Cii)** shows data for heart rate. In panels **(Ai,Aii)**, baseline conditions are indicated by “c”. In panels **(Ai)** through **(Bii)**, “h1” indicates hypertension 1, and “h2” indicates hypertension 2. The third circle indicates the baseline conditions. The heart rate period under hypertension (both h1 and h2) is shown as an “x” in panels **(Ci,Cii)**.

The dependence of systolic arterial blood pressure (P_*SAT*_) and flow (Q_*SAT*_) on model parameters is shown in Figure 3. In comparison to baseline conditions, hypertension 1 (realistic hypertension, abbreviated as h1) increased P_*SAT*_ by 17% due to small vessel resistance increase (Figure 3Ai) and by 30% due to aortic stiffness increase (Figure 3Aii). In contrast, hypertension 2 (abbreviated as h2) increased the P_*SAT*_ by 156% due to an increased small vessel resistance (Figure 3Ai), and by 84% due to augmented aortic stiffness (Figure 3Bi). Increasing R_*SAR*_ and R_*SCP*_ also reduced the systolic arterial flow, Q_*SAT*_ (Figure 3Aii). When compared to the stiffness of the systematic veins and the pulmonary veins, the aortic stiffness was the dominant factor regulating P_*SAT*_ (Figure 3Bi) and Q_*SAT*_ (Figure 3Bii) under realistic hypertension conditions. Finally, an increase of heart rate (i.e., reduction of pacing period) caused both systolic blood pressure and flow to rise marginally (Figures 3Ci,Cii).

### Effects of Hypertension Inducing Factors on Systemic Vein Flow

**FIGURE 4:**
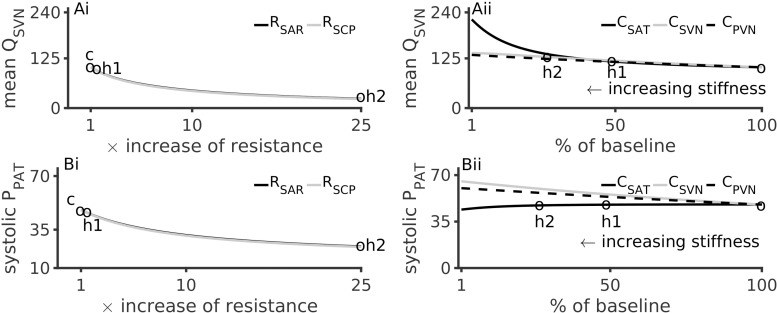
Effects of arterial hypertension inducing factors on mean systemic venous flow, Q_*SVN*_, and pulmonary systolic pressure, P_*PAT*_. **(Ai)**: Reduction of Q_*SVN*_ (representing urine flow) with increase of peripheral resistances. **(Aii)**: Increase of Q_*SVN*_ with respect to vessel stiffness (inverse of compliances, C_*SAT, SVN, PVN*_). **(Bi)**: P_*PAT*_ reduces with increase of systemic arteriolar resistances. **(Bii)**: Effects of arterial stiffness on P_*PAT*_. In all panels, the pressure and flow under control (c), hypertension 1 (h1) and hypertension 2 (h2) conditions are indicated by circles.

Figure 4 illustrates the effects of altered microvascular resistances on systemic vein flow, Q_*SVN*_ and pulmonary artery pressure, P_*PAT*_. Under hypertension 1, Q_*SVN*_ reduced by 10%, and under hypertension 2 it reduced by over 80% (Figure 4Ai). In contrast Q_*SVN*_ increased by 4% under hypertension 1 and by 45% under hypertension 2 (Figure 4Aii). As the small vessel arteriolar resistances increased, venous flow reduced, which is in accordance with the clinical observation of low urine output during renal failure.

### Effects of Hypertension Inducing Factors on Pulmonary Circulation

Hypertension inducing factors were also found to affect pulmonary circulation. Figure 4Bi show that a 25-fold increase of peripheral microvascular (systemic arteriolar) resistances reduced the pulmonary pressure (P_*PAT*_) by 75%. A similar but smaller effect was observed due to hypertension 1 conditions. Increased aortic stiffening (4-fold reduction in C_*SAT*_) lead to a modest reduction of P_*PAT*_ (Figure 4Bii).

### Alterations to Cardiac Stroke Work

The pressure-volume loops under four conditions are shown in Supplementary Figure S5. The four conditions were baseline, increased R_*SAR*_, reduced C_*SAT*_ (i.e., increased aortic stiffness), and an increased heart rate, where hypertension 2 parameter values for R_*SAR*_ and C_*SAT*_ were used. When heart rate was increased, the area of the pressure-volume loop was significantly smaller than that of that in the baseline case. The pressure-volume loops for increased R_*SAR*_ and for decreased C_*SAT*_ bound a similar area as in the baseline case, they were, however, elongated. Hypertension 1 showed similar but less marked alterations of the pressure-volume loops (not shown).

### Hemodynamic Effects of Simultaneous Ventricle Hypertrophy and Increased Peripheral Microvascular Resistance

**FIGURE 5:**
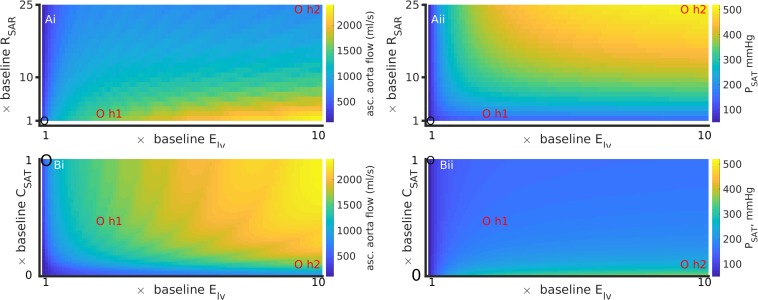
Simultaneous effects of left ventricular hypertrophy (E_*lv*_) and peripheral resistance (R_*SAR*_) **(Ai, Aii)**; and E_*lv*_ and aortic stiffness (inverse of C_*SAT*_) **(Bi, Bii)** on systematic arterial (aortic) flow and pressure, at a high heart rate (period = 0.5 s). Left column **(Ai,Bi)** shows data for ascending aorta flow (Q_*AO*_). Right column **(Aii,Bii)** shows data for aortic pressure (P_*SAT*_). Control values in each panel are shown by a black circle, parameter values for hypertensive states 1 and 2 are indicated by red circles. Also see Supplementary Figure S6 for effects on pulmonary circulation. The results for a heart rate of 1 Hz are presented in Supplementary Figures S8, S9.

The patient’s comorbid condition of the simultaneous cardiac hypertrophy and systemic small vessel blocking was mimicked by simultaneously altering left ventricle elastance and arteriolar resistance and paced at 2 Hz (Figure 5). The effect of hypertension 2 was more pronounced as compared to hypertension 1. For any given value of R_*SAR*_, the ascending aorta blood flow increased due to increased hypertrophy. The aortic flow at baseline R_*SAR*_ was 450 ml/s, at hypertension 1 was 1250 ml/s, and at hypertension 2 was 920 ml/s (Figure 5Ai). The aortic pressure also increased from 100 mmHg (baseline) to 125 mmHg at hypertension 1, and to 201 mmHg under hypertension 2 (Figure 5Aii). Bearing in mind that both definitions involve simultaneous increase of R_*SAR*_ and E_*LV*_, the model suggests that hypertension promotes increased flow as well as pressure in the aorta. The increased amount of fluid at a higher pressure will exert increased radial tension on the aortic wall leading it to dilatate (Mayet and Hughes, 2003). A cross section from one panel of Figure 5 is further illustrated in Supplementary Figure S10 for clarity of reading the color variations. The pulmonary pressure was largely unaffected (Supplementary Figure S6). Whereas stroke work increased due to the hypertrophy alone, an increased peripheral resistance blunted the effect of the hypertrophy on stroke work (Supplementary Figure S6). Similar model response was observed at a more physiological pacing rate (1 s heart period) as shown in Supplementary Figures S8, S9.

### Hemodynamic Effects of Simultaneous Ventricle Hypertrophy and Arterial Stiffening

The patient’s comorbid condition of simultaneous cardiac hypertrophy and ascending aortic dilatation was mimicked by simultaneously altering left ventricle elastance (E_*lv*_) and arterial stiffness (inverse of compliance, C_*SAT*_) (Figures 5Bi,Bii). Figure 5 has been constructed using compliance parameter rather than stiffness to avoid conversion scaling and maintain a straightforward reading of the model parameters. An increase of ventricular hypertrophy simultaneous to stiffening caused a twofold increase of ascending aorta blood flow (Figure 5Bi). The corresponding changes in aortic pressure were an increase by 1.5 fold (hypertension 1) and by 4 fold (hypertension 2). Taken together, stiff large arteries along with hypertrophy promoted increased aortic flow and pressure, which is likely to increase radial tension causing dilatation. The effects of stiffening on pulmonary artery pressure (Supplementary Figure S6) appear to be secondary to those effected by increase of systemic small vessel resistance or aortic stiffening. In contrast to the effects of thrombosis (Figure 4), arterial stiffening was seen to increase stroke work done by the heart which was exacerbated by hypertrophy (Supplementary Figure S6). Supplementary Figure S9 shows model response at heart period of 1 s.

### Dynamic Sensitivity Analysis

**FIGURE 6:**
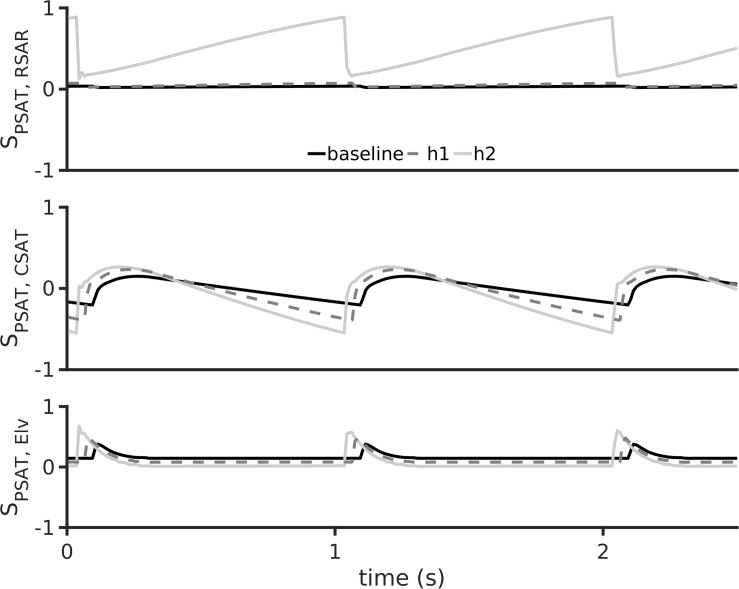
Dynamic sensitivity of aortic pressure (P_*SAT*_) to arterial hypertension inducing parameters at a heart rate period of 1 s. The parameters considered were systemic small vessel resistance (R_*SAR*_, top row), aortic stiffness (inverse of aortic compliance C_*SAT*_, middle row), and left ventricular elastance (E_*lv*_, bottom row). In all panels, black lines show baseline model, dashed gray lines show h1 data, and gray lines show h2 data.

Sensitivity of P_*SAT*_ to parameters R_*SAR*_, C_*SAT*_, and E_*lv*_ is shown in Figure 6. Under baseline conditions, the aortic pressure, P_*SAT*_, has a small sensitivity to the peripheral resistance, R_*SAR*_ (Figure 6, top panel). Under the hypertension 1 conditions, the sensitivity of P_*SA*__*T*_ to R_*SAR*_ remained unaltered, but increased appreciably under hypertension two conditions. This may indicate that treatment of peripheral resistance under severe hypertension conditions may be beneficial. As shown in Figure 6, middle panel, P_*SAT*_ is sensitive to arterial stiffness under both diseased conditions. Thus, there may be significant advantage in treatment of arterial stiffness to alleviate arterial hypertension. It appears that systemic arterial pressure is also positively sensitive to cardiac elastance (Figure 6, bottom panel), which thus making it a therapeutic target. It should be noted that the sensitivity of the arterial pressure to the systematic arterial (aortic) compliance is the largest under hypertension 1 conditions. Treatment of small vessel resistance may be less important than treatment of E_*lv*_ and C_*SAT*_.

## Conclusion

### Clinical Conclusion

Systolic arterial blood pressures of the patient was high virtually at birth but recovered upon anti-hypertensive therapy and some recovery of renal function (Figure 1). The patient had a high heart rate (Supplementary Figure S7) and was volume overloaded. The patient’s heart rate increased pathologically, instead of decreasing which is expected for normal pediatric development. In addition, the patient was underweight (Supplementary Figure S7). These observations lead us to believe that the acute kidney injury, in the form of renal vein thrombosis, had an undesirable impact on the patient’s whole-body hemodynamics. Echocardiograph revealed that the patient’s ascending aorta dilatated (Figure 2) in the early days of her life and failed to recover completely after a 6-month period. Thus, aortic dilatation is a clinically measurable quantity that is linked to multiple causal factors of hypertension highlighted in this study.

### Mechanistic Lumped Parameter Modeling Conclusion

The indicative modeling results presented in this study confirmed multiple pathophysiological mechanisms that may underlie the hypertension. Firstly, the primary cause of hypertension (i.e., high arterial systolic blood pressure) could be identified as aortic stiffness. In our model left ventricular hypertrophy and increased resistance in the systematic arteries were also associated with arterial hypertension. It may be noted that an increase of the microvasculature resistances increased systolic pressure but decreased systolic flow in the systematic arteries (Figures 3Ai,Aii). In contrast, increased stiffness increased the systolic pressure and flow (Figures 3Bi,Bii). In either case, the model suggests that the same systemic mechanisms that induce hypertension also affect the pulmonary circulation. Increased microvasculature thrombosis, i.e., increased systemic arteriolar resistance, reduced both the pulmonary artery pressure as well as flow in the systematic veins (Figures 4Ai,Bi). However, sensitivity analysis revealed that aortic stiffening may be more significant than the effects of small vessel thrombosis. Further, the major hypertension inducing mechanisms may also be responsible for severely reducing venous blood flow, which may provide some explanation for the observed severely reduced urine in the patient. Although hypertension increases the amplitude of the arterial pressure’s waveform, it may not promote better cardiac function under pathological conditions due to loss of blood movement. Increased left ventricle hypertrophy, simulated by increasing the maximal left ventricle elastance (E_*lv,max*_) in this study, increased the pressure of blood ejected from the left ventricle. At higher pacing rates, the increased pressure causes blood to flow at a higher pressure into the aorta, simultaneous to high systemic resistance (Figure 5). At more physiological rates, similar results were obtained under comparatively larger hypertension causing parameter values (Supplementary Figure S8). As the model shows, ventricular hypertrophy promotes an increased pumping pressure as well as large blood flow out of the ventricle, leading to the conclusion that ventricular hypertrophy is a relevant cause of aortic dilatation. Importantly, it indicates that other clinical measurements such as diastolic values of pressures are valuable and may assist in diagnosis and treatment design.

## Model Limitations

Although biophysically detailed, the presented phenomenological results must be considered cautiously bearing model limitations in mind. For efficacy, 0D models should be further informed by 3D simulations and experimental-clinical data to produce physiological flow rates and pressures. The Windkessel parameters can be estimated using non-invasive and routine clinical measurements such as systolic and diastolic pressures, as well as heart rates. 0D models can also be calibrated for patient-specific geometries and measurements (Lambermont et al., 1998; Molino et al., 1998; Kind et al., 2010; Ismail et al., 2013). However, the calculation of velocity fields, wall shear will require 1D and potentially 3D simulations. It can be appreciated that spatially extended simulations of any biological phenomena are computationally intensive, see a past high performance computing study (Kharche et al., 2008).

Due to the limited amount of data regarding the child’s weight and heart rate (Supplementary Figure S7), both remain be quantitatively incorporated into the model. It can be appreciated that model response was obtained at two key heart rates (0.5 and 1 Hz) which may partially reflect the patient heart rates in Supplementary Figure S7. Further, the presented model’s response appears to be overall increasing (see Supplementary Figure S10), especially in the parameter set explored, which implies that its response to heart rates at different parametric values may be inferred. This is especially true since clinical decision making, at least in this case, requires only an indicative (phenomenological) result rather than quantitative accuracy. Inclusion of the weight into the model requires significant model development and is beyond the scope of this study. A more important manner of personalization that we will perform in future studies is by estimating cardiac volume using dynamic MRI or echocardiography, which will provide a direct input to the model in terms of cardiac elastance estimates. In contrast to neonate end diastolic left ventricular volumes being approximately 40–100 mL (Dyar et al., 2011; Lytrivi et al., 2011), the presented model uses volumes between 50–150 mL (Supplementary Figure S5). In the present study, whereas the cardiac volume could be informed using data in the wider literature, such a scaling would require adjustments to most model parameters. Whereas such extensive parameter identification is out of scope of the present study, our future *de novo* descriptions will incorporate extant data. As the peripheral resistance is clinically defined as the ratio of blood pressure and cardiac output, routine clinical assessments can provide estimates of cardiac output may permit estimation of the peripheral resistance (Lee et al., 2013). Availability continuous arterial pressure recordings as well as ultrasound of the aorta may provide information regarding blood vessel stiffness (Mackenzie et al., 2002). Aortic flow may also be assessable from peripheral pulse measurements using pulse propagation modeling as performed by Poleszczuk et al. (2018). While we have identified that these are some of the limitations of the presented work, the reader can appreciate that the effective clinical treatment of the patient based on the clinicians extensive experience was paramount, and acquiring research quality data was not targeted. We reiterate that our future studies will benefit from personalization of the mechanistic modeling.

In our model, the global stress and strain of the heart tissue was not estimated. Other models can incorporate shapes of blood vessels and cardiac walls by adapting signals from mechanically loading the tissues (Arts et al., 2005). Further, the deviations of variables from their target value due to observed clinical changes of geometry may quantitatively affect the presented results (Arts et al., 2012). Suitable future developments are being implemented to also account for regurgitation. In future studies, our model will be augmented to include more sophisticated mechanisms (Arts et al., 2005). Whereas limitations have been identified, the results presented in this work will remain unaffected in the qualitative information they have provided.

The presented model allowed identification of cardiac hypertrophy as a cause of the dilatation. However, there are a number of other causes for aortic dilatation (Aalberts et al., 2008) which will improve the model’s capabilities. Further development of models that incorporates cellular biology and anatomy may assist to predict relevant quantities such as aortic diameter grown based on remodeling (Hao et al., 2017) may find clinical applications. Combined with the low dimensional models (Korakianitis and Shi, 2006b; Duanmu et al., 2018), the interplay between peripheral hypertension and the ascending aorta diameters can be better explored. Our study highlights the data inputs to mathematical models, which can then provide insights and predictions. The availability of quantitative imaging (Dinsmore, 1990; Nienaber et al., 1994; Baliyan et al., 2018) and blood (Vezzoli et al., 2017) data may permit hybrid 0D–3D, or fully 3D physiologically detailed modeling. However, our present qualitative results will be unaffected in potential higher dimensional investigations.

The model does not encompass autoregulation and baroreflex mechanisms as used by Heldt et al. (2010). These important feedback and feedforward mechanisms may further explain the time course of the return of ascending aortic diameters to normal values upon treatment of the hypertension. The model will benefit from incorporating these mechanisms in our future studies. To this end, we are undertaking a clinical-imaging-computational study that will permit further development and importantly personalization of such models, as done in Duanmu et al. (2018). Furthermore, the model is not designed to simulate essential hypertension which will require further model development. Among other factors, neurological factors may also play a significant role under pathological conditions and especially in hypertension (Averina et al., 2015).

At present, we assessed the suitability of the model to reproduce, at least qualitatively, our clinical observations. As the model was found to be able to simulate the effects of thrombosis and hypertension, we intent further developing it to include more physiological features that will assist our future clinical-imaging research. Finally, quantitative *in silico* study of the complex bio-chemistry and gene regulation is beyond the scope of the modeling but should be addressed to further improve our clinical practices.

## Clinical Discussion

To the best of our knowledge, this is the first clinical study demonstrating an association between acute ascending aortic dilatation and arterial hypertension in neonates. Further, the aortic dilatation could be partly corrected by treatment of arterial stiffness.

Whereas we saw that arterial hypertension caused ascending aorta dilatation in neonates, other factors have been identified (Zarate et al., 2015). The presence of a bicuspid aortic valve is known to be the most prominent cause of pediatric aortic dilatation as a congenital defect (Tadros et al., 2009). However, we did not find evidence of clinically significant congenital defects in our patient. Genetic testing also ruled out connective tissue disorders such as Marfan’s or Loyes-Dietz syndrome (Meester et al., 2017), which implied that clinically significant structural remodeling was largely absent. However, we acknowledge that a full recovery of the ascending aortic diameters could not be achieved. A full recovery may not have been possible due to abnormal fetal vascular growth (Persson and Buschmann, 2011), as a consequence of the complicated pregnancy when the child suffered anoxia. Vessel wall remodeling due to the blood vessel dilatation (aneurysm) and associated sub-optimal mechanical function (Tang et al., 2005), in the presence of the systemic hypertension may also arrest the reversibility of the dilatation. We appreciate that the above-mentioned factors were simultaneous to the patient’s physiological post-natal growth, which we will address in future studies.

In the context of this clinical-modeling study, we need to discuss the physiology of the arterial tree to understand the possible mechanisms that can lead to transient proximal aortic dilatation. The arterial tree has two purposes: first it acts as a conduit relying on width of the arteries and systemic vascular resistance, the second purpose is to ensure continuous steady flow in systole and diastole by dampening pressure oscillations cause by the intermittent ejection from the left ventricle (Windkessel effect). The arterial blood pressure wave is amplified as it travels away from the heart due to the summation of forward and reflective waves, the reflective waves are higher further away from the proximal aorta (London and Pannier, 2010). The way that the arterial tree adapts to increased blood pressure is by increasing the stiffness and thickness of the arteries, with a gradient from proximal (more compliant and thinner wall) to distal (increased stiffness and thicker walled), which is due to the differences in the medial layer of the aorta, composed by bands of elastin filaments, collagen fibers, and smooth muscle cells; there is a higher concentration of elastin filaments and less vascular smooth muscle cells the thoracic aorta and less elastin filaments and more vascular smooth muscle cells in the abdominal aorta (Wolinsky and Glagov, 1969; Humphrey and Na, 2002). The smooth muscle cells have an important role as they contract when subjected to increased blood pressure (Bayliss, 1902), furthermore they become hypertrophied in chronic arterial hypertension to maintain normal wall stress (Berry and Greenwald, 1976). These proprieties ensure that the wall stress remains normal. Using the Laplace law we can calculate the wall stress by the formula: *H* = *P*×*r*/(2*T*), where *H* is the circumferential wall stress, *P* is the transmural pressure gradient, *r* in the radius and *T* is the wall thickness; therefore, for a given transmural pressure and aortic diameter there must be a proportional wall thickness to maintain normal wall stress.

In our case the acute elevation of arterial hypertension without a compensatory thickening of the wall, would increase the wall stress and produce aortic dilatation, this was reverted when the blood pressure was controlled and was not likely related to the kidney injury. This is different in children and young adults with arterial hypertension where prevalence of ascending aortic dilatation is 10% which is usually associated with increased ventricular mass, and it appears to be a chronic phenomenon (Totaro et al., 2016). In summary, this neonate with acute kidney injury and subsequent chronic kidney disease had normal aortic dimensions after birth and developed significant aortic dilation within 1 month due to arterial hypertension. Upon aggressive control of the hypertension, the aortic dilation normalized. This case evidences that arterial hypertension must be added to the causes of aortopathy in chronic kidney disease pediatric patients.

## Model Discussion

**FIGURE 7:**
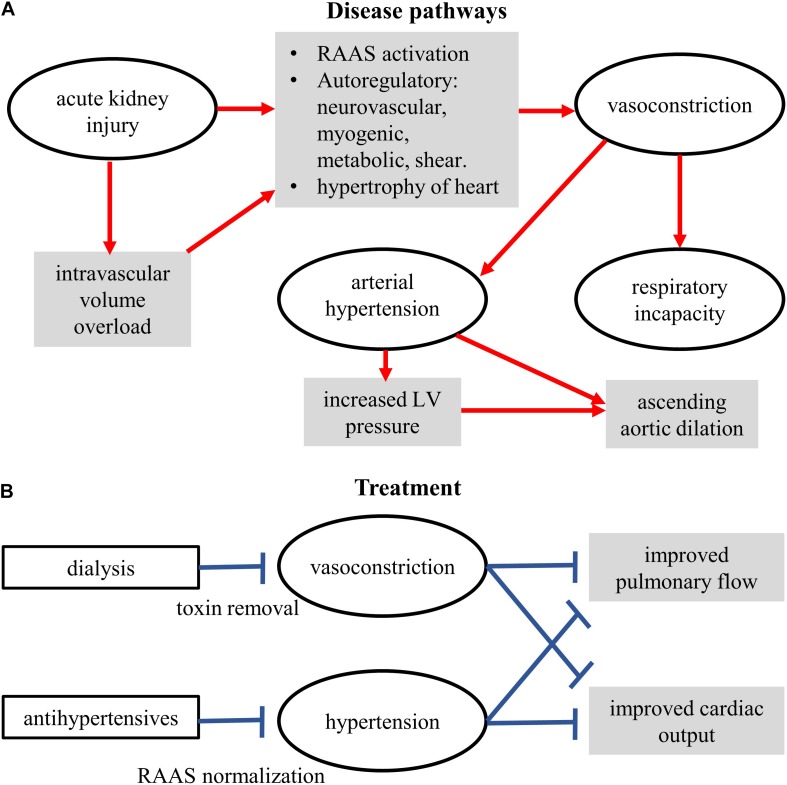
Schematic of the conceptual mechanisms for action of disease **(A)** and treatments **(B)**. Some components were measured clinically, some demonstrated by the model, some yet to be studied.

The presented model permits exploration of overall blood flow and pressure dynamics in the whole body. Although relatively simple as compared to other contemporary models, it has a large number of variables and parameters that have been validated using clinical data. It assisted us to highlight the most effective parts of the anti-hypertensive therapy with and without thrombosis. Therefore, as a first recourse, use of the Shi model is justified. However, in future studies, more detailed representations (0D or otherwise) of organ and whole-body vasculature will be implemented to further permit personalization of the computer models to clinical service. This is relevant as several clinically identified mechanisms are known to be present (Figure 7).

From a clinical treatment design perspective, the model confirms our diagnosis and provides further indications. The modeling results firstly confirm the clinical diagnosis of arterial stiffness, cardiac hypertrophy, and augmented peripheral resistance as a cause of hypertension (Figure 3). Increase of these two factors also explained, to a certain extent, the reduction of observed urine (i.e., as seen in the reduced venous blood flow, Figure 4). Within the confines of the model, the simulation indicates that apart from treating arterial stiffness and peripheral resistance, cardiac hypertrophy may also be considered as a treatment option (Figure 5). As indicated by the sensitivity analysis of Figure 6, peripheral resistance becomes more relevant in the severe hypertension cases. Whereas lacking in the model, peripheral resistance, blood vessel stiffness, and cardiac hypertrophy are linked and may not impact arterial blood pressure independently of each other. The current version of the model also shows (Supplementary Figure S6) that the resistance and stiffness probably affect pulmonary circulation, which indicates that some form of ventilation support may benefit the very young patient. Whereas the model is limited (see Section 7), its further development will permit a more detailed treatment design prior to subjective diagnosis-treatment action. Conversely, we are now equipped with an understanding of how to deploy virtual systems in gaining insights into disease and treatments.

A spectrum of other computational studies have explored the hemodynamics of hypertension to uncover relationships among vascular structure and hemodynamic observables. Similar to presented Figures 3–5, Liang et al. (2018) have simulated hypertension by altering cardiac elastance, arterial stiffness, and small vessel resistance. They found that increased small vessel resistance and arterial stiffness promoted hypertension in the form of elevated systolic arterial pressure, which is consistent with our findings (see Figure 5 and Supplementary Materials). Their sensitivity analysis shows that systolic arterial pressure was most sensitive to microvascular resistance followed by arterial stiffness and cardiac elastance, whereas we found that systolic arterial pressure was most sensitive to arterial stiffness, a s shown in our Figure 6. We appreciate that the study by Liang et al. (2018) uses advanced multi-scale modeling with a spectrum of inter-related mechanisms, an aspect that will inform our future work. The most generalized hemodynamic descriptions in the human is the Guyton model (Montani and Van Vliet, 2009). A recent study that used the Guyton model demonstrated the possible analysis in virtual patients (Moss et al., 2012). Whereas the Guyton model incorporates significant more detail, they representative analysis shows similar qualitative information as found in this study in regard to the relationship between microvascular resistance, arterial stiffness, cardiac elastance, and hypertension. Conversely, mathematical descriptions of pulmonary hypertension by others have shown the associated deleterious effects on arterial and cardiac hemodynamics (Lumens and Delhaas, 2012) which supports the findings of this study (Supplementary Figure S6).

Despite the straightforward nature of the model used, this study has provided insights into the mechanisms of the observed hypertension. It has provided a plausible explanation for the observed aortic dilatation, as well as providing potential predictions.

## Take Home Messages

### Clinical

1. To the best of our knowledge, this is the first case report in newborn with arterial hypertension secondary to acute kidney injury who developed significant aortic dilation in month 1 of her birth, which was reversible up on stringent blood pressure therapy.
2. Ascending aorta dilatation may develop very quickly in a patient with arterial hypertension. The dilatation should be explored as a potential diagnostic marker of arterial hypertension, especially since it can be examined non-invasively using echocardiograms as done in the clinical part of this study.

### Model

(i) Arterial stiffness should be treated for most effective treatment of hypertension.

(ii) In the treatment of hypertension, treatment of micro-vascular resistance and cardiac hypertrophy is also important.

(iii) Cardiac hypertrophy is a factor that acts along with microvascular resistance and aortic stiffness to promote a dilated ascending aorta.

## Author’s Note

For reproducibility and future development, our codes and anonymized data used in this study are available from the open source repository: https://github.com/mccsssk2/Arterial-Hypertension.git.

## Data Availability Statement

All datasets generated for this study are included in the manuscript and the Supplementary Files.

## Author Contributions

LA-D, GF, and CM designed and performed the clinical diagnostic exams, and delivered the therapy. SK and CM designed the modeling study. SK implemented the computer model codes, designed the simulation experiments, and performed the high throughput computing. AK assisted in model testing and annotating. SK and AK constructed the modeling figure drafts. LA-D and GF wrote the clinical draft sections. SK, AK, and BS wrote the modeling draft sections. All authors revised and approved the manuscript.

## Conflict of Interest

The authors declare that the research was conducted in the absence of any commercial or financial relationships that could be construed as a potential conflict of interest.

## References

[B1] Aalberts J. J. van den Berg M. P. Bergman J. E. du Marchie Sarvaas G. J. Post J. G. van Unen H. (2008). The many faces of aggressive aortic pathology: loeys-dietz syndrome. *Neth. Heart J.* 16 299–304. 10.1007/bf03086168 18827873 PMC2553155

[B2] Abdelhammed A. I. Smith R. D. Levy P. Smits G. J. Ferrario C. M. (2005). Noninvasive hemodynamic profiles in hypertensive subjects. *Am. J. Hypertens.* 18 51S–59S. 15752933 10.1016/j.amjhyper.2004.11.043

[B3] Arts T. Delhaas T. Bovendeerd P. Verbeek X. Prinzen F. W. (2005). Adaptation to mechanical load determines shape and properties of heart and circulation: the CircAdapt model. *Am. J. Physiol. Heart Circ. Physiol.* 288 H1943–H1954. 15550528 10.1152/ajpheart.00444.2004

[B4] Arts T. Lumens J. Kroon W. Delhaas T. (2012). Control of whole heart geometry by intramyocardial mechano-feedback: a model study. *PLoS Comput. Biol.* 8:e1002369. 10.1371/journal.pcbi.1002369 22346742 PMC3276542

[B5] Averina V. A. Othmer H. G. Fink G. D. Osborn J. W. (2015). A mathematical model of salt-sensitive hypertension: the neurogenic hypothesis. *J. Physiol.* 593 3065–3075. 10.1113/jphysiol.2014.278317 26173827 PMC4532527

[B6] Baliyan V. Verdini D. Meyersohn N. M. (2018). Noninvasive aortic imaging. *Cardiovasc. Diagn. Ther.* 8 S3–S18. 29850415 10.21037/cdt.2018.02.01 PMC5949599

[B7] Bayliss W. M. (1902). On the local reactions of the arterial wall to changes of internal pressure. *J. Physiol.* 28 220–231. 10.1113/jphysiol.1902.sp000911 16992618 PMC1540533

[B8] Beard D. A. Britten R. Cooling M. T. Garny A. Halstead M. D. Hunter P. J. (2009). CellML metadata standards, associated tools and repositories. *Philos. Trans. A Math. Phys. Eng. Sci.* 367 1845–1867. 10.1098/rsta.2008.0310 19380315 PMC3268215

[B9] Berry C. L. Greenwald S. E. (1976). Effects of hypertension on the static mechanical properties and chemical composition of the rat aorta. *Cardiovasc. Res.* 10 437–451. 10.1093/cvr/10.4.437 947333

[B10] Boegehold M. A. Huffman L. J. Hedge G. A. (1991). Peripheral vascular resistance and regional blood flows in hypertensive dahl rats. *Am. J. Physiol.* 261 R934–R938. 1928439 10.1152/ajpregu.1991.261.4.R934

[B11] Caruso M. V. Gramigna V. Renzulli A. Fragomeni G. (2016). Computational analysis of aortic hemodynamics during total and partial extracorporeal membrane oxygenation and intra-aortic balloon pump support. *Acta Bioeng. Biomech.* 18 3–9. 27840434

[B12] Cohen-Solal A. Caviezel B. Himbert D. Gourgon R. (1994). Left ventricular-arterial coupling in systemic hypertension: analysis by means of arterial effective and left ventricular elastances. *J. Hypertens.* 12 591–600. 7930560

[B13] Dinsmore R. E. (1990). Noninvasive imaging of thoracic aortic disease. *Curr. Opin. Radiol.* 2 595–601. 2204399

[B14] Duanmu Z. Yin M. Fan X. Yang X. Luo X. (2018). A patient-specific lumped-parameter model of coronary circulation. *Sci. Rep.* 8:874. 10.1038/s41598-018-19164-w 29343785 PMC5772042

[B15] Dyar D. A. Fripp R. R. Printz B. F. (2011). Normal values for left ventricular volume in infants and young children: questions for the authors. *J. Am. Soc. Echocardiogr.* 24:933. 10.1016/j.echo.2011.05.007 21664799

[B16] Evangelista A. Flachskampf F. A. Erbel R. Antonini-Canterin F. Vlachopoulos C. Rocchi G. (2010). Echocardiography in aortic diseases: EAE recommendations for clinical practice. *Eur. J. Echocardiogr.* 11 645–658. 10.1093/ejechocard/jeq056 20823280

[B17] Ferretti F. Saltelli A. Tarantola S. (2016). Trends in sensitivity analysis practice in the last decade. *Sci. Total Environ.* 568 666–670. 10.1016/j.scitotenv.2016.02.133 26934843

[B18] Flachskampf F. A. Wouters P. F. Edvardsen T. Evangelista A. Habib G. Hoffman P. (2014). Recommendations for transoesophageal echocardiography: EACVI update 2014. *Eur. Heart J. Cardiovasc. Imag.* 15 353–365. 10.1093/ehjci/jeu015 24554131

[B19] Fleming S. Thompson M. Stevens R. Heneghan C. Pluddemann A. Maconochie I. (2011). Normal ranges of heart rate and respiratory rate in children from birth to 18 years of age: a systematic review of observational studies. *Lancet* 377 1011–1018. 10.1016/S0140-6736(10)62226-X 21411136 PMC3789232

[B20] Flynn J. T. Mitsnefes M. Pierce C. Cole S. R. Parekh R. S. Furth S. L. (2008). Blood pressure in children with chronic kidney disease: a report from the chronic kidney disease in Children study. *Hypertension* 52 631–637. 10.1161/HYPERTENSIONAHA.108.110635 18725579 PMC3136362

[B21] Galesic K. Brkljacic B. Sabljar-Matovinovic M. Morovic-Vergles J. Cvitkovic-Kuzmic A. Bozikov V. (2000). Renal vascular resistance in essential hypertension: duplex-doppler ultrasonographic evaluation. *Angiology* 51 667–675. 10959519

[B22] Gul R. Bernhard S. (2015). Parametric uncertainty and global sensitivity analysis in a model of the carotid bifurcation: identification and ranking of most sensitive model parameters. *Math. Biosci.* 269 104–116. 10.1016/j.mbs.2015.09.001 26367184

[B23] Gul R. Schütte C. Bernhard S. (2016). Mathematical modeling and sensitivity analysis of arterial anastomosis in the arm. *Appl. Math. Modell.* 40 7724–7738. 10.1016/j.apm.2016.03.041

[B24] Hao W. Gong S. Wu S. Xu J. Go M. R. Friedman A. (2017). A mathematical model of aortic aneurysm formation. *PLoS One* 12:e0170807. 10.1371/journal.pone.0170807 28212412 PMC5315396

[B25] Hardt S. E. Just A. Bekeredjian R. Kubler W. Kirchheim H. R. Kuecherer H. F. (1999). Aortic pressure-diameter relationship assessed by intravascular ultrasound: experimental validation in dogs. *Am. J. Physiol.* 276 H1078–H1085. 10.1152/ajpheart.1999.276.3.H1078 10070094

[B26] Heldt T. Mukkamala R. Moody G. B. Mark R. G. (2010). CVSim: an open-source cardiovascular simulator for teaching and research. *Open Pacing Electrophysiol. Ther. J.* 3 45–54. 21949555 PMC3178445

[B27] Humphrey J. D. Na S. (2002). Elastodynamics and arterial wall stress. *Ann. Biomed. Eng.* 30 509–523. 10.1114/1.1467676 12086002

[B28] Ismail M. Wall W. A. Gee M. W. (2013). Adjoint-based inverse analysis of windkessel parameters for patient-specific vascular models. *J. Comput. Phys.* 244 113–130. 10.1016/j.jcp.2012.10.028

[B29] Kaddourah A. Uthup S. Madueme P. O’Rourke M. Hooper D. K. Taylor M. D. (2015). Prevalence and predictors of aortic dilation as a novel cardiovascular complication in children with end-stage renal disease. *Clin. Nephrol.* 83 262–271. 10.5414/CN108489 25816808 PMC4535175

[B30] Kahan T. (1998). The importance of left ventricular hypertrophy in human hypertension. *J. Hypertens. Suppl.* 16 S23–S29. 9855028

[B31] Kharche S. Ludtke N. Panzeri S. Zhang H. (2009). A global sensitivity index for biophysically detailed cardiac cell models: a computational approach. *LNCS* 28:10.

[B32] Kharche S. Seemann G. Margetts L. Leng J. Holden A. V. Zhang H. (2008). Simulation of clinical electrophysiology in 3D human atria: a high-performance computing and high-performance visualization application. *Concurr. Comp. Exp.* 20 1317–1328. 10.1002/cpe.1332

[B33] Kharche S. R. So A. Salerno F. Lee T. Ellis C. Goldman D. (2018). Computational assessment of blood flow heterogeneity in peritoneal dialysis patients’ cardiac ventricles. *Front. Physiol.* 9:511. 10.3389/fphys.2018.00511 PMC5968396 29867555

[B34] Kharche S. R. Stary T. Colman M. A. Biktasheva I. V. Workman A. J. Rankin A. C. (2014). Effects of human atrial ionic remodelling by beta-blocker therapy on mechanisms of atrial fibrillation: a computer simulation. *Europace* 16 1524–1533. 10.1093/europace/euu084 25085203 PMC4640177

[B35] Kharche S. R. Vigmond E. Efimov I. R. Dobrzynski H. (2017). Computational assessment of the functional role of sinoatrial node exit pathways in the human heart. *PLoS One* 12:e0183727. 10.1371/journal.pone.0183727 28873427 PMC5584965

[B36] Kind T. Faes T. J. Lankhaar J. W. Vonk-Noordegraaf A. Verhaegen M. (2010). Estimation of three- and four-element windkessel parameters using subspace model identification. *IEEE Trans. Biomed. Eng.* 57 1531–1538. 10.1109/TBME.2010.2041351 20172779

[B37] Korakianitis T. Shi Y. (2006a). A concentrated parameter model for the human cardiovascular system including heart valve dynamics and atrioventricular interaction. *Med. Eng. Phys.* 28 613–628. 10.1016/j.medengphy.2005.10.004 16293439

[B38] Korakianitis T. Shi Y. (2006b). Numerical simulation of cardiovascular dynamics with healthy and diseased heart valves. *J. Biomech.* 39 1964–1982. 10.1016/j.jbiomech.2005.06.016 16140309

[B39] Lambermont B. D’Orio V. Gerard P. Kolh P. Detry O. Marcelle R. (1998). Time domain method to identify simultaneously parameters of the windkessel model applied to the pulmonary circulation. *Arch. Physiol. Biochem.* 106 245–252. 10.1076/apab.106.3.245.4378 10099721

[B40] Lee Q. Y. Redmond S. J. Chan G. Middleton P. M. Steel E. Malouf P. (2013). Estimation of cardiac output and systemic vascular resistance using a multivariate regression model with features selected from the finger photoplethysmogram and routine cardiovascular measurements. *Biomed. Eng. Online* 12:19. 10.1186/1475-925X-12-19 23452705 PMC3649882

[B41] Liang F. Guan D. Alastruey J. (2018). Determinant factors for arterial hemodynamics in hypertension: theoretical insights from a computational model-based study. *J. Biomech. Eng.* 140:031006. 10.1115/1.4038430 29131886

[B42] Liu Z. R. Ting C. T. Zhu S. X. Yin F. C. (1989). Aortic compliance in human hypertension. *Hypertension* 14 129–136. 10.1161/01.hyp.14.2.129 2759675

[B43] London G. M. Pannier B. (2010). Arterial functions: how to interpret the complex physiology. *Nephrol. Dial. Transplant.* 25 3815–3823. 10.1093/ndt/gfq614 20947536

[B44] Lumens J. Delhaas T. (2012). Cardiovascular modeling in pulmonary arterial hypertension: focus on mechanisms and treatment of right heart failure using the CircAdapt model. *Am. J. Cardiol.* 110 39S–48S. 10.1016/j.amjcard.2012.06.015 22921031

[B45] Lytrivi I. D. Bhatla P. Ko H. H. Yau J. Geiger M. K. Walsh R. (2011). Normal values for left ventricular volume in infants and young children by the echocardiographic subxiphoid five-sixth area by length (bullet) method. *J. Am. Soc. Echocardiogr.* 24 214–218. 10.1016/j.echo.2010.12.002 21281912

[B46] Mackenzie I. S. Wilkinson I. B. Cockcroft J. R. (2002). Assessment of arterial stiffness in clinical practice. *QJM* 95 67–74. 10.1093/qjmed/95.2.67 11861952

[B47] Marsden A. Moghadam M. E. (2015). Multiscale modeling of cardiovascular flows for clinical decision support. *Appl. Mech. Rev.* 67:030804.

[B48] Marsden A. L. (2013). Simulation based planning of surgical interventions in pediatric cardiology. *Phys. Fluids* 25:101303. 10.1063/1.4825031 24255590 PMC3820639

[B49] Mayet J. Hughes A. (2003). Cardiac and vascular pathophysiology in hypertension. *Heart* 89 1104–1109. 10.1136/heart.89.9.1104 12923045 PMC1767863

[B50] Meester J. A. N. Verstraeten A. Schepers D. Alaerts M. Van Laer L. Loeys B. L. (2017). Differences in manifestations of marfan syndrome, ehlers-danlos syndrome, and loeys-dietz syndrome. *Ann. Cardiothorac. Surg.* 6 582–594. 10.21037/acs.2017.11.03 29270370 PMC5721110

[B51] Meyers A. Joyce K. Coleman S. M. Cook J. T. Cutts D. Ettinger de Cuba S. (2013). Health of children classified as underweight by CDC reference but normal by WHO standard. *Pediatrics* 131 e1780–e1787. 10.1542/peds.2012-2382 23690515

[B52] Milan A. Tosello F. Naso D. Avenatti E. Leone D. Magnino C. (2013). Ascending aortic dilatation, arterial stiffness and cardiac organ damage in essential hypertension. *J. Hypertens.* 31 109–116. 10.1097/HJH.0b013e32835aa588 23221933

[B53] Molino P. Cerutti C. Julien C. Cuisinaud G. Gustin M. P. Paultre C. (1998). Beat-to-beat estimation of windkessel model parameters in conscious rats. *Am. J. Physiol.* 274 H171–H177. 10.1152/ajpheart.1998.274.1.H171 9458865

[B54] Montani J. P. Van Vliet B. N. (2009). Understanding the contribution of Guyton’s large circulatory model to long-term control of arterial pressure. *Exp. Physiol.* 94 382–388. 10.1113/expphysiol.2008.043299 19286638

[B55] Moss R. Grosse T. Marchant I. Lassau N. Gueyffier F. Thomas S. R. (2012). Virtual patients and sensitivity analysis of the Guyton model of blood pressure regulation: towards individualized models of whole-body physiology. *PLoS Comput. Biol.* 8:e1002571. 10.1371/journal.pcbi.1002571 22761561 PMC3386164

[B56] Nienaber C. A. von Kodolitsch Y. Brockhoff C. J. Koschyk D. H. Spielmann R. P. (1994). Comparison of conventional and transesophageal echocardiography with magnetic resonance imaging for anatomical mapping of thoracic aortic dissection. A dual noninvasive imaging study with anatomical and/or angiographic validation. *Int. J. Cardiac. Imag.* 10 1–14. 10.1007/bf01151576 8021526

[B57] Persson A. B. Buschmann I. R. (2011). Vascular growth in health and disease. *Front. Mol. Neurosci.* 4:14. 10.3389/fnmol.2011.00014 21904523 PMC3160751

[B58] Pettersen K. H. Bugenhagen S. M. Nauman J. Beard D. A. Omholt S. W. (2014). Arterial stiffening provides sufficient explanation for primary hypertension. *PLoS Comput. Biol.* 10:e1003634. 10.1371/journal.pcbi.1003634 24853828 PMC4031054

[B59] Poleszczuk J. Debowska M. Dabrowski W. Wojcik-Zaluska A. Zaluska W. Waniewski J. (2018). Patient-specific pulse wave propagation model identifies cardiovascular risk characteristics in hemodialysis patients. *PLoS Comput. Biol.* 14:e1006417. 10.1371/journal.pcbi.1006417 30216341 PMC6157900

[B60] Rabkin S. W. Janusz M. T. (2013). Aortic wall stress in hypertension and ascending thoracic aortic aneurysms: implications for antihypertensive therapy. *High Blood Pressure Cardiovasc. Prev.* 20 265–271. 10.1007/s40292-013-0026-z 24092647

[B61] Saltelli A. (2002). Sensitivity analysis for importance assessment. *Risk Anal.* 22 579–590. 10.1111/0272-4332.00040 12088235

[B62] Schindelin J. Arganda-Carreras I. Frise E. Kaynig V. Longair M. Pietzsch T. (2012). Fiji: an open-source platform for biological-image analysis. *Nat. Methods* 9 676–682. 10.1038/nmeth.2019 22743772 PMC3855844

[B63] Serne E. H. de Jongh R. T. Eringa E. C. IJzerman R. G. Stehouwer C. D. (2007). Microvascular dysfunction: a potential pathophysiological role in the metabolic syndrome. *Hypertension* 50 204–211. 10.1161/hypertensionaha.107.089680 17470716

[B64] Shi Y. Lawford P. Hose R. (2011). Review of zero-D and 1-D models of blood flow in the cardiovascular system. *Biomed. Eng. Online* 10:33. 10.1186/1475-925X-10-33 21521508 PMC3103466

[B65] Tadros T. M. Klein M. D. Shapira O. M. (2009). Ascending aortic dilatation associated with bicuspid aortic valve: pathophysiology, molecular biology, and clinical implications. *Circulation* 119 880–890. 10.1161/circulationaha.108.795401 19221231

[B66] Tang P. C. Coady M. A. Lovoulos C. Dardik A. Aslan M. Elefteriades J. A. (2005). Hyperplastic cellular remodeling of the media in ascending thoracic aortic aneurysms. *Circulation* 112 1098–1105. 10.1161/circulationaha.104.511717 16116068

[B67] Totaro S. Rabbia F. Milan A. Urbina E. M. Veglio F. (2016). Aortic root dilatation in the children and young adults: prevalence, determinants, and association with target organ damage. *J. Am. Soc. Hypertens.* 10 782–789. 10.1016/j.jash.2016.07.008 27637377

[B68] Vezzoli M. Bonardelli S. Peroni M. Ravanelli M. Garrafa E. (2017). A simple blood test, such as complete blood count, can predict calcification grade of abdominal aortic aneurysm. *Int. J. Vasc. Med.* 2017:1370751. 10.1155/2017/1370751 28948050 PMC5602620

[B69] Warren A. E. Boyd M. L. O’Connell C. Dodds L. (2006). Dilatation of the ascending aorta in paediatric patients with bicuspid aortic valve: frequency, rate of progression and risk factors. *Heart* 92 1496–1500. 10.1136/hrt.2005.081539 16547208 PMC1861027

[B70] Wolinsky H. Glagov S. (1969). Comparison of abdominal and thoracic aortic medial structure in mammals. Deviation of man from the usual pattern. *Circ. Res.* 25 677–686. 10.1161/01.res.25.6.677 5364644

[B71] Zarate Y. A. Sellars E. Lepard T. Carlo W. F. Tang X. Collins R. T. II (2015). Aortic dilation in pediatric patients. *Eur. J. Pediatr.* 174 1585–1592. 10.1007/s00431-015-2575-8 26070999

