# Supplementary material for: Arterial Hypertension and Unusual Ascending Aortic Dilatation in a Neonate With Acute Kidney Injury: Mechanistic Computer Modeling
Source: Front Physiol. 2019 Nov 8;10:1391. doi: 10.3389/fphys.2019.01391 (PMC6856675; doi:10.3389/fphys.2019.01391)
Supplement: Supplementary file 1 [file Data_Sheet_1.pdf]

SUPPLEMENTARY MATERIALS

Arterial hypertension and unusual ascending aortic dilatation in a neonate with acute kidney injury: mechanistic computer modelling

Luis AltamiranoDiaz MD[1,2,3], Andrea D. Kassay BMSc[4], Baran Serajelahi PhD[6], C W McIntyre MD[4], Guido Filler MD[1,2,4,5], Sanjay R. Kharche PhD[4,5,6*]

[1]Department of Pediatrics, Schulich School of Medicine and Dentistry, Western University, London, ON, Canada. [2] Children's Health Research Institute, London, ON, Canada. [3]Pediatric Cardiopulmonary Research Laboratory, LHSC, London, ON, Canada. [4]Lawson Health Research Institute, London, ON, Canada. [5]Department of Medicine, Schulich School of Medicine and Dentistry, Western University, London, ON, Canada. [6] Department of Medical Biophysics, Western University, London, ON, Canada

Keywords: dialysis, hypertension, aortic dilatation, computer model, lumped parameter blood flow model, sensitivity analysis.

[*]**corresponding author**:

Dr. Sanjay R. Kharche Ph.D.

Research Scientist and Adjunct Professor of Medical Biophysics,

Room ELL 112, 800 Commissioners Road, Victoria Hospital,

Lawson's Health Research Institute, University of Western Ontario,

London ON, Canada, N6A 5W9.

Phone: 519-685-6047

Fax: 519-685-8072

Email: Sanjay.Kharche@lhsc.on.ca

Section S1. Supplementary Methods

Section S1.1 Detailed case report.

The patient was born at term by Caesarean section with fetal heart rate decelerations following induction of labor for premature rupture of membranes at 41 + 6 weeks of gestation. APGAR (appearance of skin color, pulse providing heart rate, grimace responses to assess reflexes, activity to assess muscle tone, and respiration assessed using breathing rate and effort) scores were 2, 7, 7 (risk of high mortality) at one, five and ten minutes, respectively (Natarajan et al., 2013). The patient required resuscitation with positive pressure ventilation. The patient developed significant hypotension and poor perfusion needing fluid bolus and vasopressors.

The patient developed acute kidney injury with oligoanuria and hematuria needing peritoneal dialysis by day 5 of life, which was continued for 9 days. This treatment was based on sudden onset anuria, macrohematuria, hypertension, bilaterally large kidneys on ultrasound with high resistive indices exceeding 1 (normal 0.7-0.8, (Bude et al., 1992)) and low blood flow in the apparent patent renal veins, as well as transient thrombocytopenia, the diagnosis of bilateral renal vein thrombosis was assumed, although no thrombus was seen in the vena cava (Resontoc & Yap, 2016). Dialysis was stopped because the patient regained sufficient urinary output.

Contrast angiography was avoided given the patient's critical status. Heparin therapy was also avoided because of concerns of intracranial bleeding. Despite the continuous dialysis treatment (hourly dwells using 2.27% physioneal at 10 mL/kg) with reasonable ultrafiltration up to 1.5 mL/kg/h, she developed arterial hypertension with blood pressures consistently above the 99th percentile. She was therefore given a 5-day course of amlodipine with good initial response, but at one month of age the blood pressure increased again to > 99th percentile (National High Blood Pressure Education Program Working Group on High Blood Pressure in & Adolescents, 2004), requiring ongoing anti-hypertensive therapy, including the addition of enalapril. Figure 1 shows systolic blood pressure in the first month of life. Echocardiogram was performed at day one of life showing normal cardiac structure and normal ascending aortic diameter, as well as two small muscular ventricular septal defects. The repeated echocardiogram at 1 month of age showed significant ascending aortic dilatation (see Figures 2). Aortic dilatation was reduced considerably by month 5. Clinically the patient did not have stigmata of Marfan's or Loeys-Dietz syndromes.

Section S1.2 ImageJ (Schindelin et al., 2012) method of aortic diameter assessment from echocardiograph images.

Echocardiography exams were performed using a commercially available echocardiographic machine Philips iE33. DICOM images were exported from the machine to USB, anonymized, and ported to users work station. Image analysis to calculate aortic diameters was performed using ImageJ-FiJi open source

software. The method can be summarized as the following steps.

1. The DICOM images contain 3 frames each. Using MATLAB functions, individual frames were extracted. In case an animation was exported from the 2D echocardiography machine, functions in the opensource program *ImageMagic* were used to extract all frames. In all four cases, the echo exams consisted of three heart beat data. The individual frames had the echo image, superposed with an ECG trace to assist in gating.

2. Using the ECG trace, the second heart beat between the second and third R peaks were analyzed for diameters.

3. The sequentially numbered frames were imported into ImageJ using the GUI menu.

4. The brightness and contrast were adjusted to provide a better onscreen image suitable for analysis by the expert. The minimum and maximum brightness were adjusted such that the range contained all peaks of the grayscale distribution.

5. Using information from the literature, and as shown in the inset of Figure 1B, lines were drawn and the distance in terms of pixels was obtained.

7. The resolution of pixels in the images was obtained from the 2D echocardiography machine.

6. The pixel wise distance was converted into mm units using the pixel size of dx = 0.075 mm, and dy = 0.075 mm.

Section S1.2 Modelling parameters and variables.

Table S1. The analogous metrics between the electrical domain and hemodynamics along with their typical symbols, and units in each system.

Electrical	Fluid dynamics
Voltage, V [V]	Pressure, P [mmHg]
Current, I [A]	Flow rate, Q [ml s^{-1}]
Resistance, R [Ω]	Resistance, R [mmHg s ml^{-1}]
Inductance, L [H]	Inertia or inductance, I [mmHg s^2 ml^{-1}]
Charge, Q [C]	Volume, V [ml]
Capacitance, C [F]	Compliance, C [ml $mmHg^{-1}$]

Table S2. System states and computed algebraic variables used in this study. For all other variables, see the model's original publication as well as the online code provided.

State variable (symbol)	Units	description
time, t	seconds, s	independent variable.
P_{SAT}	mmHg	systemic artery pressure.
P_{PAT}	mmHg	pulmonary artery pressure.
Q_{SAT}	ml/s	systemic artery (aorta) blood flow.
Q_{SVN}	ml/s	systemic vein (vena cava) blood flow.
Q_{AO}	ml/s	aortic root (ascending aorta) blood flow.
E_{lv}	F^{-1}	left ventricle elastance.

Table S3. Parameters used in this study. All other parameters were left unaltered, and can be found in the original model article as well as the online code provided.

Parameter	Units	Description	Values (Control, hypertension 1, hypertension 2)
R_{SAR}	mmHg s ml^{-1}	Systemic arteriolar resistance.	0.5, 0.75, 12.5
R_{SCP}	mmHg s ml^{-1}	Systemic capillary bed resistance.	0.52, 0.78, 13
C_{SAT}	ml $mmHg^{-1}$	Systemic artery (aorta) compliance.	1.6, 0.75, 0.4
C_{SVN}	ml $mmHg^{-1}$	Systemic vein (vena cava) compliance.	20.5, 9.64, 5.13
C_{PVN}	ml $mmHg^{-1}$	Pulmonary vein compliance.	20.5, 9.64, 5.13
$E_{lv, max}$	F^{-1}	Maximum value of LV elastance.	2.5, 5, 25

Section S2. Supplementary Results

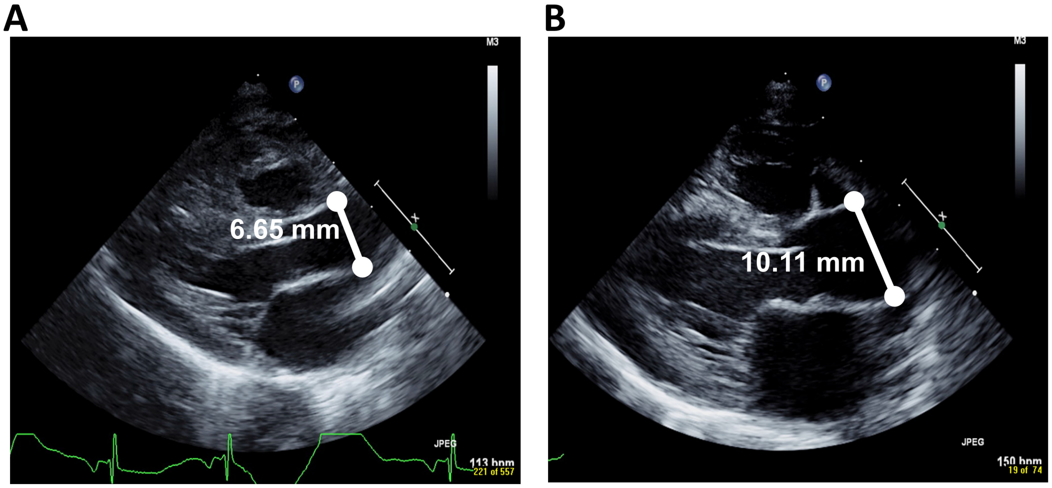

Figure S1. Representative images of the echocardiography of the ascending aorta. On day 1, the exam average ascending aortic diameter was found to be 0.65 cm. The average diameter dilated to over 1.011 cm by day 30.

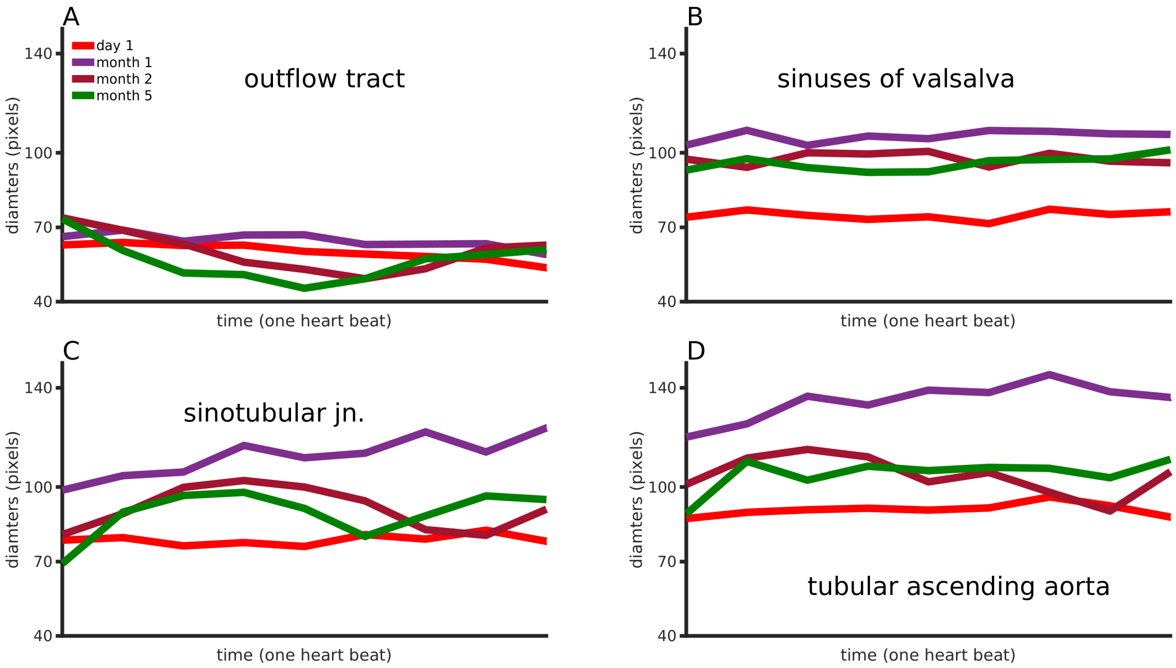

Figure S2. Aortic diameters over the course of one heart beat. The means of above data are shown in the main manuscript, Figure 2.

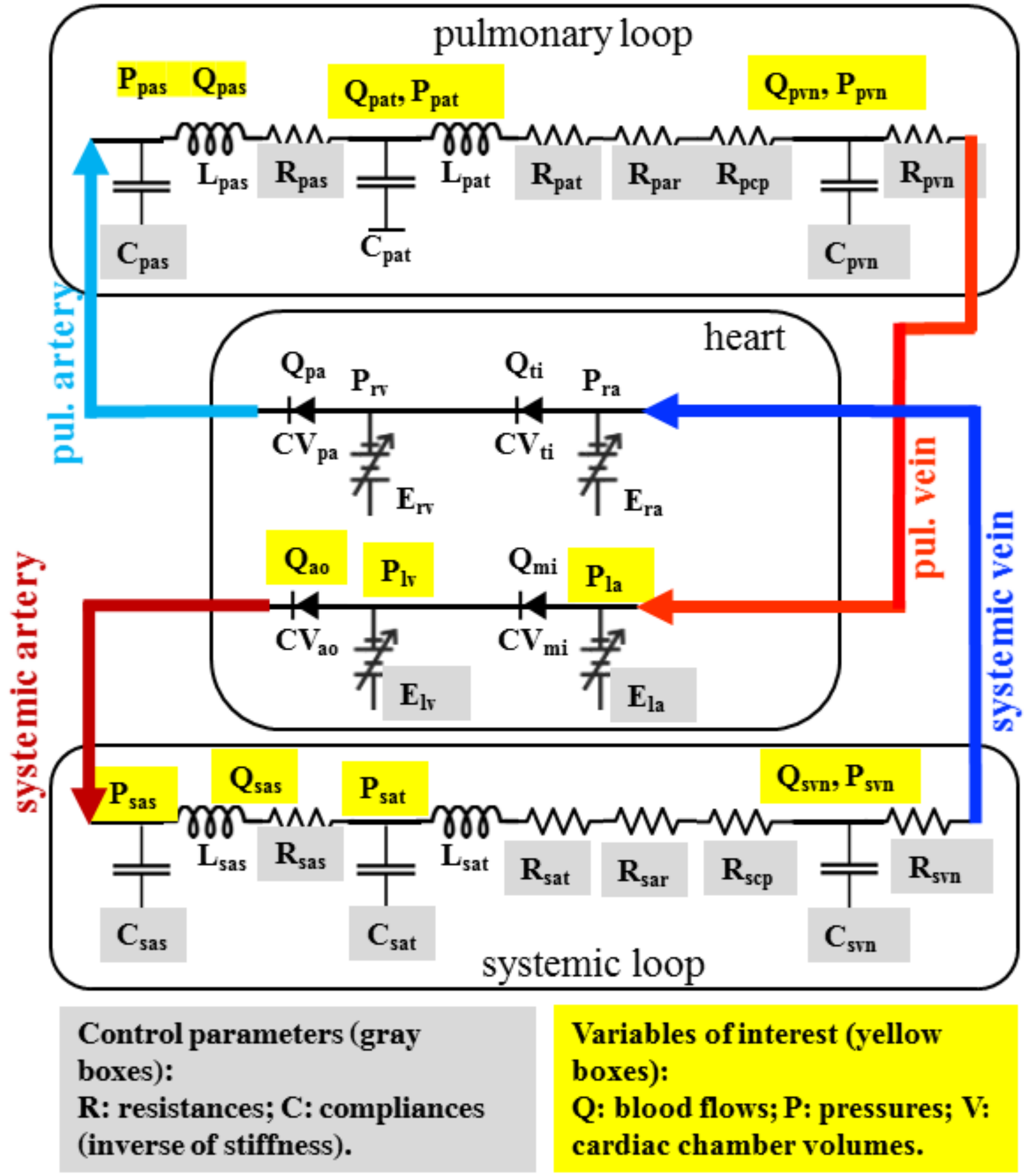

Figure S3. Schematic diagram of the model used in this study (Korakianitis & Shi, 2006). Parameters are shown in gray boxes. The variables in yellow boxes were recorded to construct modeling results.

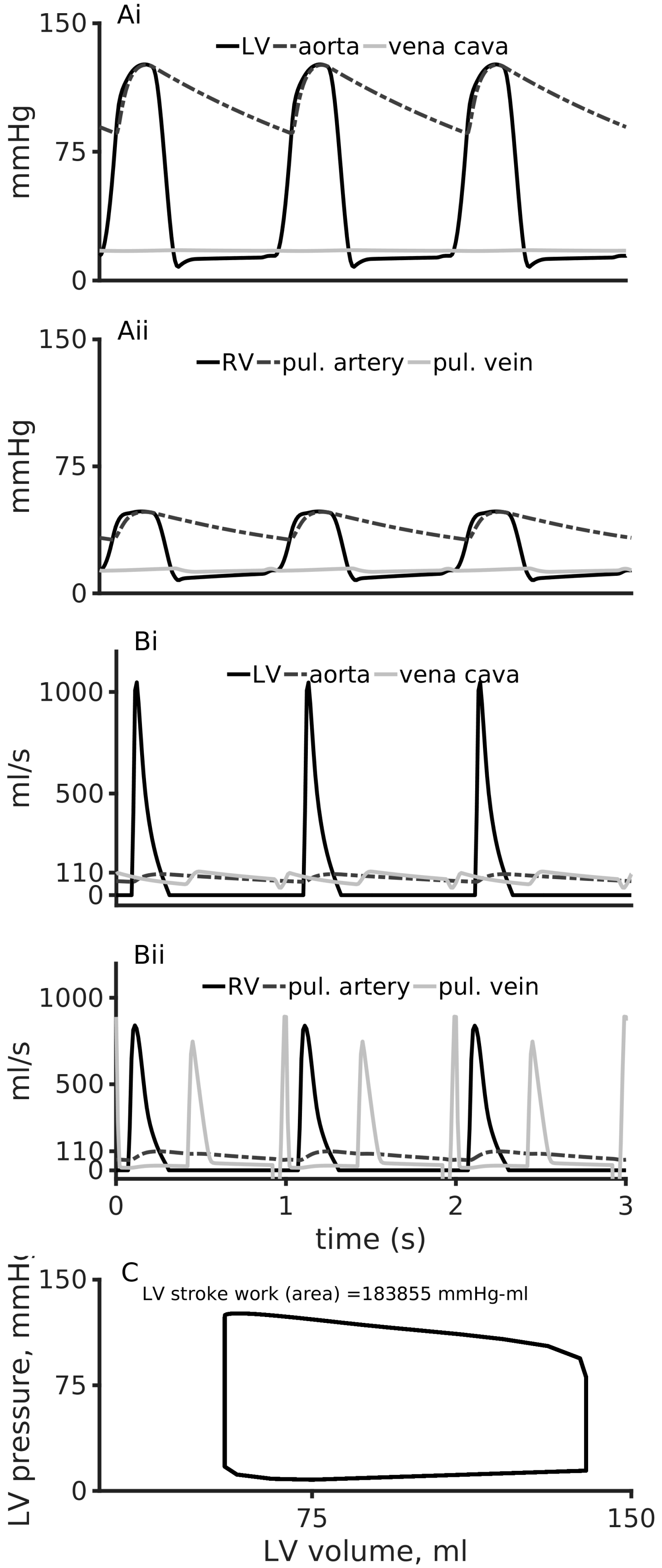
Ai
LV aorta vena cava
150
75
0
mmHg
Aii
RV pul. artery pul. vein
150
75
0
mmHg
Bi
LV aorta vena cava
1000
500
110
0
ml/s
Bii
RV pul. artery pul. vein
1000
500
110
0
ml/s
0
1
2
3
time (s)
C
LV stroke work (area) =183855 mmHg-ml
150
75
0
LV pressure, mmHg
75
150
LV volume, ml

Figure S4. Main pressures and blood flows in the baseline model. Ai. Pressures in left ventricle (black line), systemic artery (aorta, black dotted line), and systemic vein (vena cava, gray line). Aii. Pressures in right ventricle (black line), pulmonary artery (black dotted line), and pulmonary vein (gray line). Bi. Blood flow in left ventricle (black line), systemic artery (aorta, black dotted line), and systemic vein (vena cava, gray line). Bii. Blood flow in right ventricle (black line), pulmonary artery (black dotted line), and pulmonary vein (gray line). C. Left ventricle pressure-volume loop and baseline stroke work (area inside pressure-volume loop).

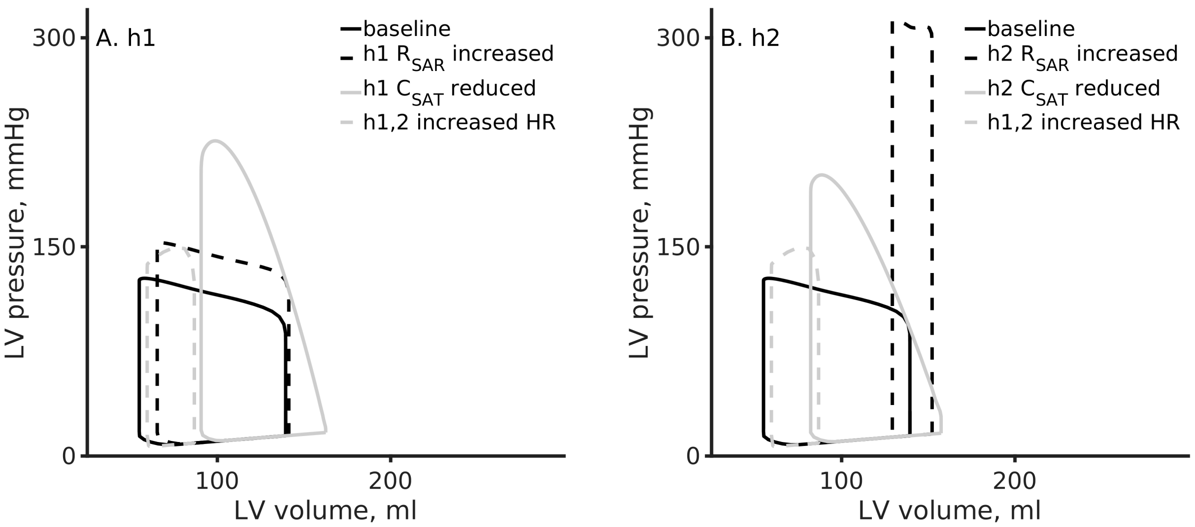

Figure S5. Pressure-volume curves under literature informed hypertension (h1, panel A) and exaggerated parameter values hypertension (h2, panel B) conditions. In both panels the black solid line represents baseline model, the dashed black line shows augmented R_{SAR}, and the gray line shows increased aortic stiffness (reduced compliance) conditions. The dashed gray lines in both panels show data for increased heart rate for both h1 and h2 conditions.

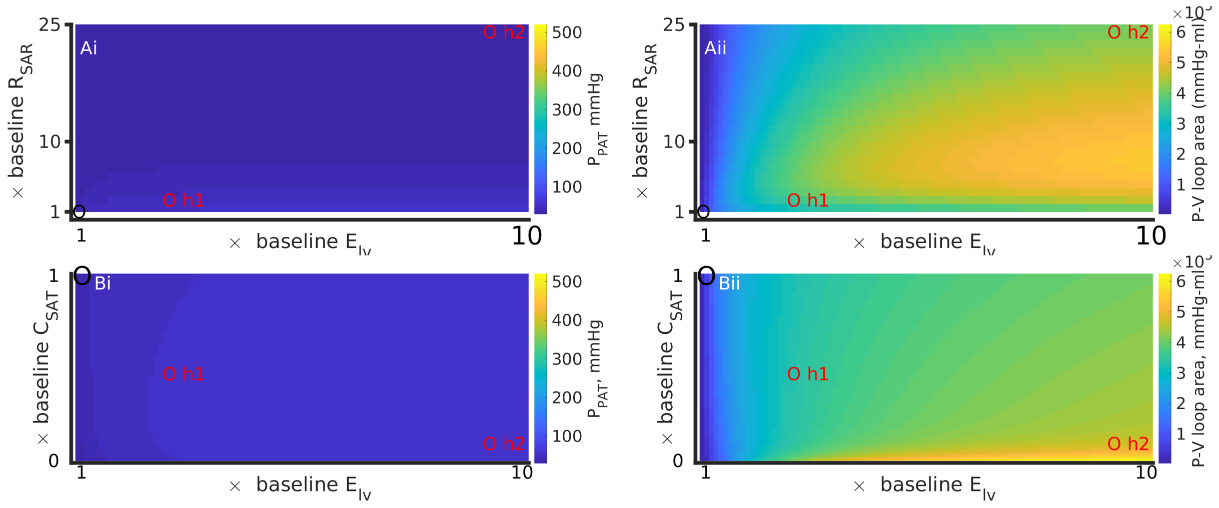

Figure S6. Simultaneous effects of left ventricular hypertrophy (E_{lv}), peripheral resistance (R_{SAR}), and aortic stiffness (inverse of C_{SAT}) on pulmonary pressure and stroke work, at a high heart rate (period = 0.5 s). Left column shows data for P_{PAT}, and right column shows data for stroke work (area of P-V loop). Black circles indicate control (baseline) values, red circles indicate parameter values for hypertensive states h1 and h2.

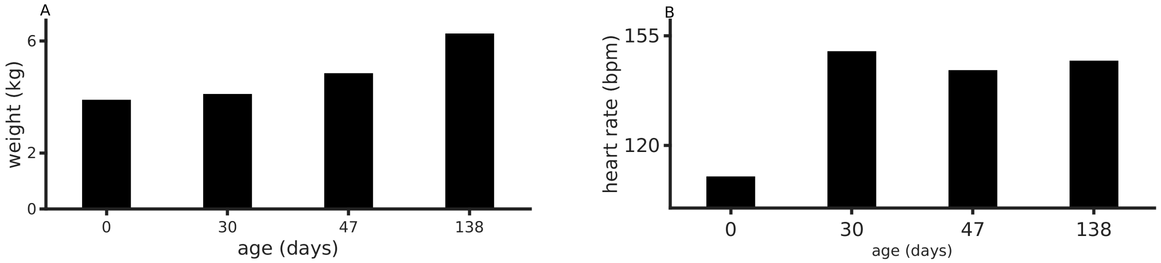

Figure S7. Patient weight and heart rate over 138 days. A: Weight. B: Heart rate.

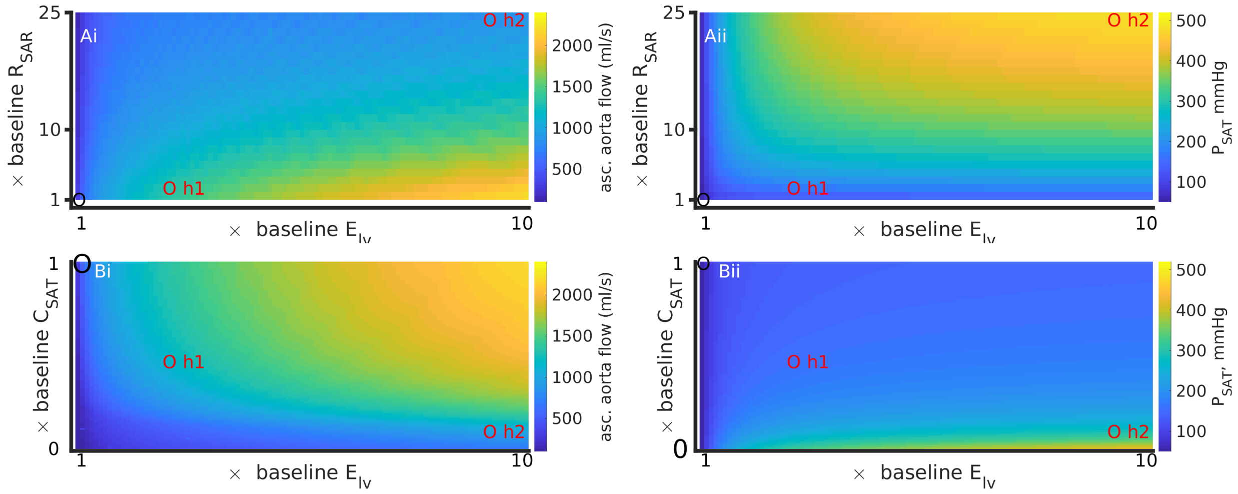

Figure S8. Simultaneous effects of left ventricular hypertrophy (E_{lv}), peripheral resistance (R_{SAR}), and aortic stiffness (inverse of C_{SAT}) on systematic arterial (aortic) flow and pressure, at a lower more physiological heart rate (period = 1 s). Left column shows data for ascending aorta flow (Q_{AO}). Right column shows data for aortic pressure (P_{SAT}). Control values in each panel are shown by a black circle, parameter values for hypertensive states h1 and h2 are indicated by red circles. Also see Figures 5 (main manuscript), S6 (above), and S9 (below).

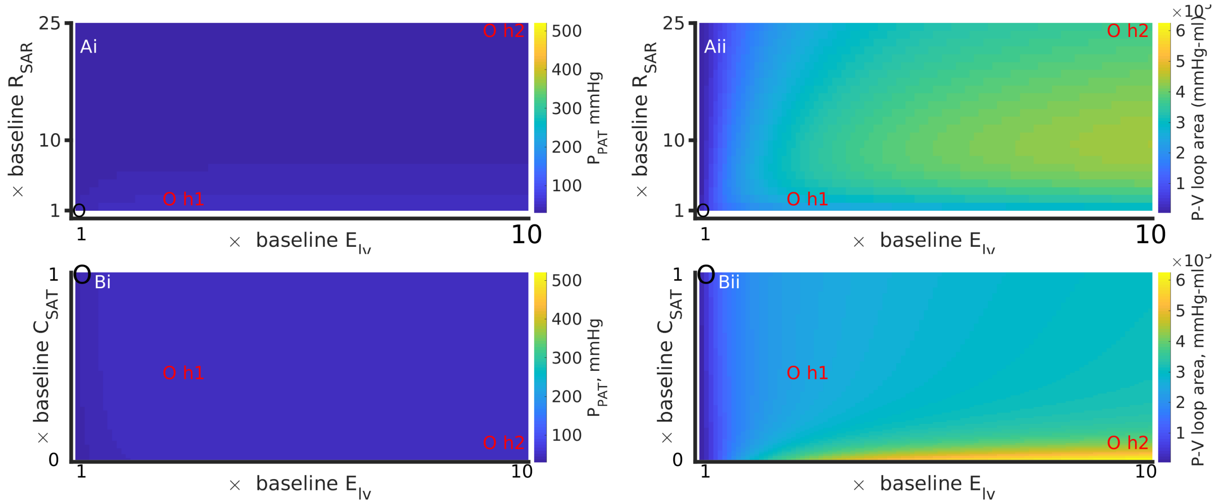

Figure S9. Simultaneous effects of left ventricular hypertrophy (E_{lv}), peripheral resistance (R_{SAR}), and aortic stiffness (inverse of C_{SAT}) on pulmonary pressure and stroke work, at a lower more physiological heart rate (period = 1 s). Left column shows data for pulmonary arterial pressure (P_{PAT}). Right column shows data for the area of the pressure volume loop (P-V loop area). Control values in each panel are shown by a black circle, parameter values for hypertensive states h1 and h2 are indicated by red circles.

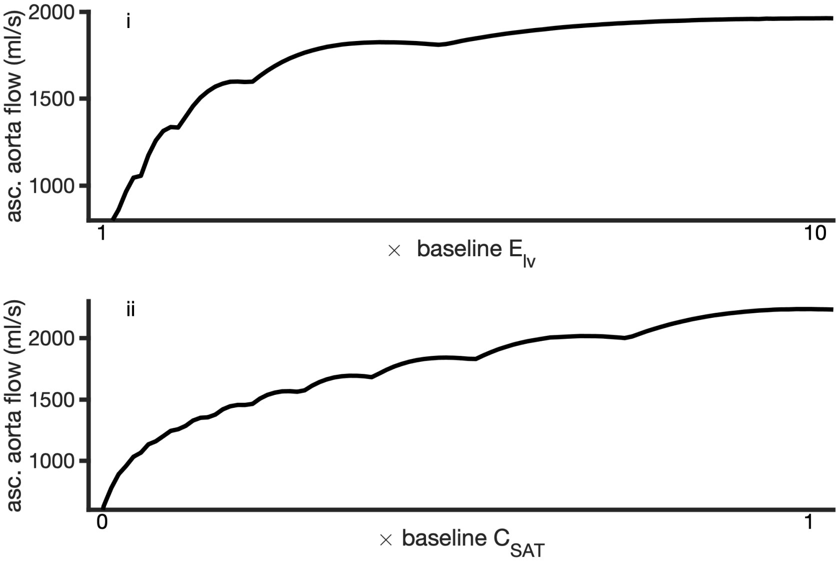

Figure S10. Representative examples of model behavior with respect to ascending aortic flow for E_{lv} ranging from 1 to 10-fold of its baseline value (top) and C_{SAT} ranging from 0 to 1-fold of its baseline value (bottom). The top panel shows the change in aortic flow with increasing E_{lv} for C_{SAT} at 0.5 of its baseline value. The bottom panel shows the change in aortic flow with increasing C_{SAT} (decreasing stiffness) for at 5 fold of its baseline value. In both panels the heart rate is 2 Hz. See also Figure 5.

References

Bude RO, DiPietro MA, Platt JF, Rubin JM, Miesowicz S & Lundquist C. (1992). Age dependency of the renal resistive index in healthy children. *Radiology* **184,** 469-473.

Korakianitis T & Shi Y. (2006). A concentrated parameter model for the human cardiovascular system including heart valve dynamics and atrioventricular interaction. *Medical engineering & physics* **28,** 613-628.

Natarajan G, Shankaran S, Laptook AR, Pappas A, Bann CM, McDonald SA, Das A, Higgins RD, Hintz SR & Vohr BR. (2013). Apgar scores at 10 min and outcomes at 6-7 years following hypoxic-ischaemic encephalopathy. *Archives of disease in childhood Fetal and neonatal edition* **98,** F473-479.

National High Blood Pressure Education Program Working Group on High Blood Pressure in C & Adolescents. (2004). The fourth report on the diagnosis, evaluation, and treatment of high blood pressure in children and adolescents. *Pediatrics* **114,** 555-576.

Resontoc LP & Yap HK. (2016). Renal vascular thrombosis in the newborn. *Pediatr Nephrol* **31,** 907-915.

Schindelin J, Arganda-Carreras I, Frise E, Kaynig V, Longair M, Pietzsch T, Preibisch S, Rueden C, Saalfeld S, Schmid B, Tinevez JY, White DJ, Hartenstein V, Eliceiri K, Tomancak P & Cardona A. (2012). Fiji: an open-source platform for biological-image analysis. *Nature methods* **9,** 676-682.
